# High-Intensity Laser Therapy for Musculoskeletal Disorders: A Systematic Review and Meta-Analysis of Randomized Clinical Trials

**DOI:** 10.3390/jcm12041479

**Published:** 2023-02-13

**Authors:** Rubén Arroyo-Fernández, Javier Aceituno-Gómez, Diego Serrano-Muñoz, Juan Avendaño-Coy

**Affiliations:** Nursing, Physiotherapy and Occupational Therapy Department, Faculty of Physiotherapy and Nursing, University of Castilla-La Mancha, Avd. Carlos III s/n., 45071 Toledo, Spain

**Keywords:** high-intensity laser therapy, musculoskeletal disorders, meta-analysis, systematic review

## Abstract

High-intensity laser therapy (HILT) is one of the therapeutic approaches used in the treatment of musculoskeletal disorders (MSD). The main objective of this study was to examine the effectiveness of HILT for reducing pain and improving functionality in people with MSD. Ten databases were systematically searched for randomized trials published up to 28 February 2022. Randomized clinical trials (RCTs) assessing the effectiveness of HILT on MSD were included. The main outcome measures were pain and functionality. In total, 48 RCTs were included in the qualitative synthesis and 44 RCTs in the quantitative analysis. HILT showed a decrease on the pain VAS (mean difference (MD) = −1.3 cm; confidence interval (CI) 95%: −1.6 to −1.0) and an improvement in functionality (standardized mean difference (SMD) = −1.0; CI95%: −1.4 to −0.7), with low and moderate quality of evidence, respectively. A greater effect was observed when compared with control than with other conservative treatments, both on pain (χ2 = 20.6; *p* < 0.001) and functionality (χ2 = 5.1; *p* = 0.02). Differences in the effectiveness of HILT were found depending on the location (χ2 = 40.1 *p* < 0.001), with further improved functionality in MSD of the knee and shoulder. HILT is an effective treatment for improving pain, functionality, range of motion, and quality of life in people with MSD, although these findings must be treated with caution due to the high risk of bias in the studies. Further clinical trials should be well designed to lower the risk of bias.

## 1. Introduction

The prevalence of musculoskeletal disorders approaches 36% in older adults in Europe [1]. This figure increases to almost 45% in the working-age Spanish population [2], making this condition an economical and public health issue [3]. Laser therapy is a standard treatment in clinical practice for the treatment of musculoskeletal disorders [4]. Although low-power laser (Class III) therapy has been employed for more than two decades [5], high-power laser (Class IV) therapy has been implemented in the clinical setting in the last years due to its greater depth of penetration and the possibility of delivering higher doses with lower exposure times [6]. Over the last few years, clinical trials have aimed at assessing the effectiveness of this new therapy for patients with musculoskeletal disorders.

One systematic review [7] and one systematic review with meta-analysis [8] for the evaluation of high-intensity laser therapy (HILT) in musculoskeletal disorders have been published, which included trials conducted up to 17 January 2018. Of the studies found, only 12 randomized trials met the criteria for inclusion, with 6 comparing HILT versus sham stimulation and 6 versus other treatments. The large number of clinical trials published after the end of the search period of this meta-analysis, the possibility to analyze the effectiveness of HILT on other outcome variables, and the lack of an analysis of effectiveness depending on the dosage call for an update of the level of evidence of HILT for patients with musculoskeletal disorders.

The main aim of this systematic review with meta-analysis was to assess the effectiveness of HILT for improving pain and functionality in adults with musculoskeletal disorders. The secondary objectives were to evaluate the effect of HILT on the range of motion (ROM), muscle strength, and quality of life of participants, as well as the safety of the treatment. Additionally, this review conducted a comparison depending on the comparator (control or conservative treatment), dosage, follow-up period, and anatomical location of treatment application. 

## 2. Materials and Methods

This systematic review and meta-analysis followed the guidelines of Preferred Reporting Items for Systematic Reviews and Meta-Analysis (PRISMA) Statement [9] and the recommendations by The Cochrane Collaboration [10]. Its protocol was registered in PROSPERO (reference number CRD42020198663). 

### 2.1. Search Strategy

Two independent researchers (RAF and DSM) searched bibliographic references on HILT for treating musculoskeletal disorders in the following databases: Pubmed, Physiotherapy Evidence Database (PEDro), Cochrane Central Register of Controlled Trials, Web of Science, Scopus, CINAHL, Ulrich’s web, ProQuest, Google Scholar, and Dialnet. The selection of articles was completed via an inverse manual search of the references cited in the articles found (Supplementary Appendix S1). The search targeted articles in English or Spanish, without limitations in terms of gender, but only including trials conducted on adults. Articles were selected from the initial dates of the relevant databases up to 28 February 2022. 

### 2.2. Selection Criteria

Randomized clinical trials (RCTs) assessing the effectiveness of HILT on musculoskeletal disorders were included. MSD comes from damage to the musculoskeletal system involving muscles, nerves, tendons, joints, and cartilage in the upper and lower limb, neck, and lower back [8]. These studies compared the HILT intervention versus sham HILT, non-exposed control group, or other treatments (both pharmacological and non-pharmacological). The criteria for exclusion were as follows: the availability of abstracts only or conference presentations; and not reporting the dosage, application parameters, or location of the HILT intervention. Two independent researchers (RAF and DSM) selected the articles based on the inclusion and exclusion criteria, and a third researcher (JAC) intervened to reach a consensus in case of disagreement.

### 2.3. Data Extraction

Two researchers (JAG and RAF) performed the data extraction by using a chart specifically designed for this purpose that they agreed upon. A third researcher (JAC) compared both charts and presented the final data collection. The main outcome variables were pain and functionality. Pain was measured as the subjective perception of the patient reported on a visual analogue scale (VAS). Functionality was assessed from values recorded in specific scales for each region. When a trial employed several scales for measuring functionality, data were extracted only from one based on an order already stated in our protocol, which had been previously registered in PROSPERO. Additionally, adverse effects reported in the studies were recorded. The secondary variables were the ROM (degrees), quality of life based on the SF-36 scale, and muscle strength measured via dynamometry. Authors of the selected studies were contacted to obtain or clarify missing or unclear data if needed. Data available only in graphs were extracted using Graph Grabber 2.0.2 software for graph digitalization (https://www.quintessa.org/software/ (accessed on 19 September 2022)).

### 2.4. Assessment of Risk of Bias

The risk of bias was assessed based on recommendations by the Cochrane organization [10] using Review Manager (RevMan) (Computer program. Version 5.3. Copenhagen: The Nordic Cochrane Centre, the Cochrane Collaboration, 2014). Two independent reviewers (RAF and JAG) evaluated the risk of bias and a third investigator (DSM) resolved cases of disagreement. Seven items were addressed for evaluating the risk of bias, and the relevant risk was expressed in three levels (unclear, low, and high). Previously, the researchers had agreed on the following: for the item “blinding of participants”, the risk would be qualified as unclear when participants were not blinded in all groups in studies with more than two arms; and for the item “selective reporting”, studies without a registered protocol would be qualified as unclear or high risk depending on the final report. Finally, funnel plots for the main variable (pain on a VAS) were analyzed to evaluate publication bias.

### 2.5. Data Synthesis and Analysis

The inverse variance method was used for analyzing all variables (pain, functionality, ROM, strength, and quality of life). Statistical heterogeneity was evaluated using the Chi-squared test (with statistical significance set at *p* < 0.10), and heterogeneity was measured calculating I2, with 25%, 50%, and 75% representing low, moderate, and high heterogeneity, respectively [10]. Random effect and fixed effect analysis models were used when the heterogeneity I² was greater or lower than 50%, respectively. The mean difference (MD) was obtained for the pain VAS, strength, and quality of life outcomes, which were expressed on the same scale in the included studies. Average values of the VAS pain were calculated whenever this outcome was reported for different situations (at rest, during movement, etcetera). In terms of quality of life assessed via the SF-36 scale, the results were evaluated for each of its 8 items since the calculation of an overall score for this scale has not been validated [11]. The standardized mean difference (SMD) was estimated for the assessment of functionality and ROM, since their measurements vary depending on the different employed scales or different units (degrees and mm), respectively. For functionality scales where a higher score means less disability (i.e., Foot and Ankle Outcome Score and Constant Murley Score scales), this value was multiplied by −1 in order to align the direction of the effect. For the ROM variable, an analysis of the sum of all the movements (degrees) of the assessed joints was performed. Only the passive ROM was included from those studies reporting both the passive and active ROM. Confidence intervals were set at 95% (CI95%) for all variables. The analyzed results were those with the longest follow-up period for each of the included studies. 

In the case of studies with more than two arms, splitting of the shared group was applied according to the Cochrane Group Guidelines [10] to avoid double count. In addition to the global analysis, an analysis by subgroups was conducted for all variables to account for the comparator (control/other therapy). For those studies comparing HILT versus control, an analysis of the main variables (pain and functionality) was conducted by subgroups to account for the follow-up period, dosage, and anatomical location of musculoskeletal pain. In the case of studies comparing HILT versus other treatments, an analysis by subgroups was performed to account for the other therapy. The RevMan 5.4.1 software was used for the quantitative analysis. The quality of evidence was classified for each outcome as high, moderate, low, or very low following the Grades of Recommendation Assessment, Development, and Evaluation (GRADE) method [12]. 

## 3. Results

**Figure 1:**
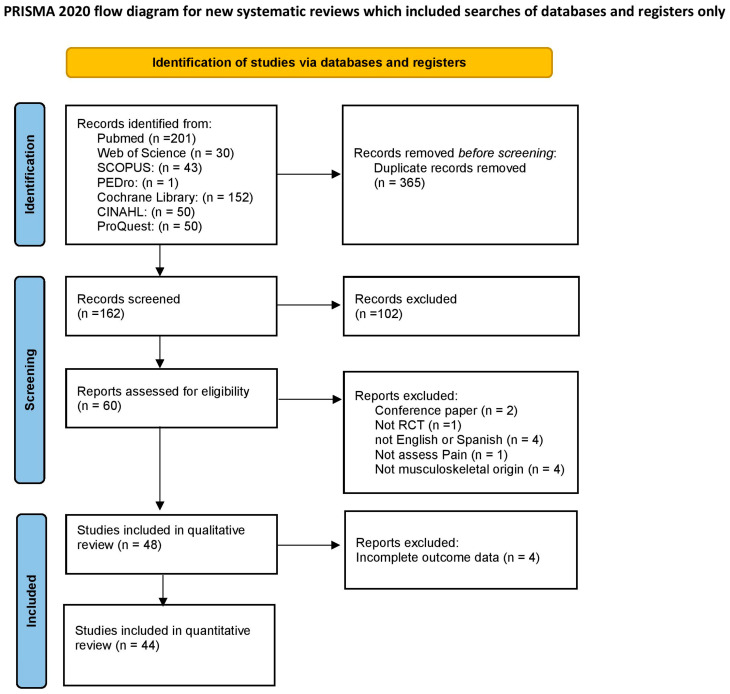
Flowchart of the systematic review and meta-analysis.

Following the removal of duplicates, 162 articles were identified as eligible, of which 102 were eliminated after reading of the title and abstract. Finally, after reading the full text, 48 RCTs were included [13,14,15,16,17,18,19,20,21,22,23,24,25,26,27,28,29,30,31,32,33,34,35,36,37,38,39,40,41,42,43,44,45,46,47,48,49,50,51,52,53,54,55,56,57,58,59,60] in the qualitative synthesis. The studies by Angelova et al. [59], Boyraz et al. [15], Akaltun et al. [58], and Hojjati et al. [32] were excluded from the pooled quantitative analysis because of reporting average values of outcomes without measures of dispersion; hence, deriving data from their report was not possible. Finally, 44 RCTs [13,14,16,17,18,19,20,21,22,23,24,25,26,27,28,29,30,31,33,34,35,36,37,38,39,40,41,42,43,44,45,46,47,48,49,50,51,52,53,54,55,56,57,60] were included in the present meta-analysis (Figure 1). Additional information was requested from the authors of eight studies [15,32,33,40,48,49,58,59] regarding characteristics of the trial or outcome data, but only one provided a reply [40].

### 3.1. Qualitative Summary of the Included Studies 

Characteristics of the included studies are shown in Supplementary Appendix S2. Overall, 14 studies compared HILT against control or sham/control [14,16,21,23,24,35,36,42,53,55,56,58,59,60], 22 studies against other interventions [17,18,19,20,26,29,30,31,33,37,38,39,40,43,44,45,46,49,50,52,54,57], and 12 against control/sham and other interventions [13,15,22,25,27,28,32,34,41,47,48,51]. The majority of trials included two arms, except for 12 three-arm studies [13,15,22,25,26,27,32,34,41,47,57,60], 2 trials with four arms [48,51], and 1 with five arms [28].

In the included RCTs, the treatment alternatives with which HILT was compared were: low-level laser therapy (LLLT) [13,28,29,32,33,34,40,45,51], transcutaneous electrical nerve stimulation (TENS)/TENS+ultrasound therapy [17,27,38,41,46,52,54], ultrasound therapy [15,26,30,44,50,57], exercising and tractions [20,25,31], bandages and orthosis [22,26,49], radiofrequency [43], and shock wave therapy [39]. Ten trials [16,17,20,24,30,31,49,50,51,59] employed HILT as the only delivered therapy, three of which compared it with placebo [16,24,59]. Taradaj et al. conducted a four-arm study that compared both HILT and LLLT against placebo [51]. Trials that delivered HILT in combination with other therapies chose to do so with exercising [13,14,15,21,23,25,27,28,29,34,35,36,38,41,42,43,44,45,46,47,48,52,53,54,55,56,58,60], ultrasound therapy [29,46], electrotherapy [29,44], thermotherapy [38,44,46], bandages and orthosis [32,33,45], manual therapy techniques [48,56], and pharmacological treatments [35,47]. 

The sample size comprised a total of 3107 participants, of which 1718 (55.3%) were women; of note, six trials [19,23,29,37,48,59] did not report the gender of subjects. The average age ranged between 28 and 64 years. Overall, losses to follow-up were identified (3.96%), 53 in the HILT group and 70 in the non-HILT group. RCTs delivered the HILT therapy for relieving pain at the following locations: temporomandibular joint [24,26,27]; neck [20,21,31,36,52,54,56]; lower back [13,15,18,19,25,30,38,46,51]; knee [34,37,39,41,42,47,58,59]; foot [40,45,53]; shoulder [14,23,35,43,44,48,50,55,60]; and elbow, wrist, or thumb [16,17,22,28,29,32,33,49,57]. Protocols were heterogeneous in terms of the reported parameters for applying HILT. Devices delivered an average power of 1–5 W in 7 studies [15,16,28,29,32,41,57], 6–10 W in 13 studies [30,31,33,38,40,42,43,44,45,49,50,54,59], 10.5 W in 16 studies [21,22,23,24,25,26,27,34,36,39,47,48,52,53,55,56], 12–30 W in 10 studies [13,14,17,18,20,35,46,58,59,60], and it was unreported in 2 studies [19,37]. Furthermore, 18 trials applied 5–10 sessions of HILT [15,18,20,28,29,31,33,35,38,40,42,43,44,45,49,50,58,59], 28 trials applied 11–20 sessions, 1 trial applied 24 sessions [13], and sessions were unreported in one trial [32]. Energetic density was ≤10 J/cm^2^ in 15 studies [19,20,21,22,25,30,34,37,39,48,50,52,53,54,56], 10–50 J/cm^2^ in 5 trials [23,29,36,46,47], 50–100 J/cm^2^ in 3 trials [31,41,51], 100–300 J/cm^2^ in 9 trials [13,14,17,18,38,40,42,57,60]. Twelve studies applied different energetic densities at different sessions [24,27,28,33,43,44,45,49,55,58,59], and four studies did not report this information [15,16,32,35].

The assessed main outcome variables were as follows: (1) pain reported on a VAS in all studies; (2) functionality (measured with the Neck and Disability Index at the neck [21,31,36,52,54]; the Oswestry Disability Index [13,15,18,25,30,38,46,51] and Roland–Morris Disability Questionnaire [25,51] at the lower back; the Shoulder Pain and Disability Index [14,23,43,44,48,60], Constant-Murley Score [14,50,55], and Disabilities of the Arm, Shoulder, and Hand [14,57] at the shoulder; the Patient-Rated Tennis Elbow Evaluation questionnaire [22] and Disabilities of the Arm, Shoulder, and Hand [33,49] at the elbow; the Boston Symptom Severity Scale [32] for carpal tunnel syndrome; Jaw Functional Limitation Scale-20 [24,26,27] for temporomandibular disorder; the Western Ontario and McMaster Universities Osteoarthritis Index [34,37,39,41,42,47,58], Time and go Test [41], 6-min walk Test [39,42], and Kujala questionnaire [42] at the knee; and the Foot and Ankle Outcome Score at the foot [45,53]). The studied secondary variables were quality of life (measured with the SF-36 questionnaire [15,21,22,33,49,53,55,60]; HRQoL: EQ-5D-3L [13]; Oral Health Impact Profile-14 [24,26,27] and Nottingham Health Profile [43]), muscle strength (grip strength measured with a hand dynamometer, key pinch strength with a pinch dynamometer and isokinetic [55]), and ROM (measured with a universal goniometer [21,23,35,46,48,54,60], CROM goniometer [36,52,56], inclinometer [21], or BROM goniometer [25] and with an electronic caliper [26] or a ruler [27]).

The follow-up periods differed among studies: an immediately post-intervention assessment was conducted in only 16 trials [13,17,20,29,30,33,34,36,37,39,45,48,50,54,56,57], at 3–9 weeks after the intervention in 20 studies [18,21,22,24,25,26,27,28,35,37,38,40,41,46,47,49,52,53,54,58], and after 10–24 weeks in 12 trials [14,15,16,23,31,32,42,43,51,55,59,60]. Assessment of adverse effects and/or complications was specifically stated in 18 of the 48 included studies [14,21,22,24,26,27,33,35,40,41,42,43,44,52,53,54,55,60], of which only 1 reported one adverse effect in a participant consisting of an allergy to HILT [60]. 

### 3.2. Risk of Bias in the Included Studies

**Figure 2:**
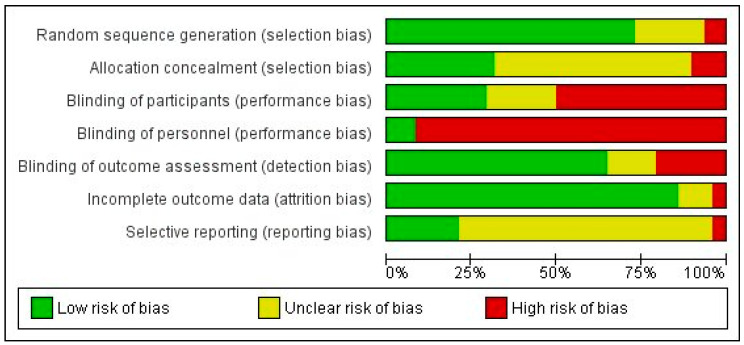
Risk of bias graph: review authors’ judgements about each risk of bias item presented as percentages across all included studies.

The two researchers that assessed the risk of bias (JAG and RAF) agreed upon 82% of the items, and disagreements were resolved by a third researcher (DSM). Figure 2 shows the summary of risk of bias for the 48 included studies. The majority of studies showed high performance bias, mainly resulting from the inability to blind the researcher and participants whenever HILT was compared with other therapy or no intervention. Low risk of bias was only found in 4 RCTs [16,36,42,56] (8.3% of included studies) for the item “blinding of personnel” and in 14 RCTs [14,16,21,23,24,25,35,36,42,53,56,58,59] (29.2%) for the item “blinding of participants”. On the contrary, 31 RCTs [13,14,16,20,21,22,23,24,26,27,28,30,32,33,35,39,41,43,44,45,46,47,48,50,51,52,53,54,55,58,60] (64.6% of included studies) were rated as having a low risk of bias regarding the blinding of outcome assessment (detection bias). 

**Figure 3:**
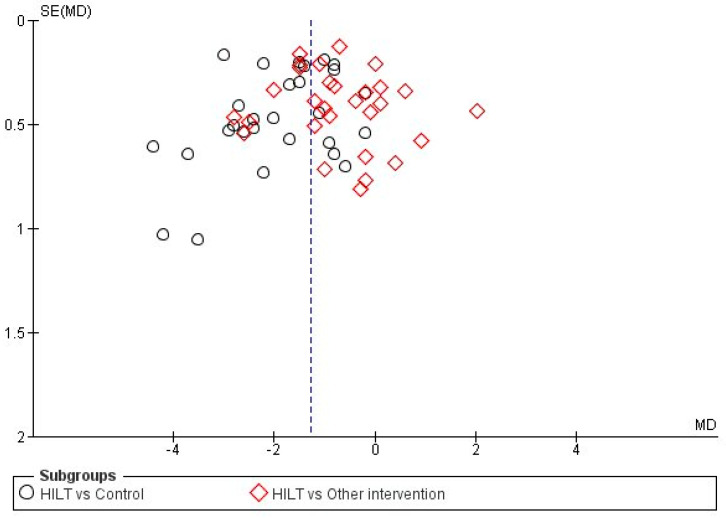
Funnel plot for the comparison of high-intensity laser therapy (HILT) vs. control/another intervention for pain outcome as measured on a VAS.

The items with the lowest risk of bias were those regarding the randomization generation and incomplete results, where high risk was observed in only three [14,51,59] and two [40,48] RCTs, respectively. Finally, the reporting bias was classified as unclear for the majority of included trials for not having previously registered their relevant protocol. The study by Naruseviciute et al. [40] was also classified as high risk for this item since their former protocol incorporated secondary variables for measuring functionality and quality of life that the final report did not include. The trial by Pekyavas et al. [48] was also rated with high risk for not reporting the VAS pain outcomes despite describing its estimation in their methods. The works by Angelova et al. [59] and Boyraz et al. [15], which were excluded from the pooled quantitative analysis due to insufficient data, were classified as having a high risk of bias due to being rated with low risk in only one and none of the assessed items, respectively. Conversely, all items were rated as low risk in the study by Cantero-Tellez et al. [16], and so were all the items but one in the trials by Nouri et al. [42] and Yesil et al. [53]. Twenty-three RCTs [14,16,21,22,23,24,27,28,30,33,35,36,39,41,42,43,45,50,53,55,56,58,60] (46.2%) presented moderate risk of bias with 4–5 items rated as low risk (see Supplementary Appendix S3 for the risk of bias for each included study). The risk of publication bias was considered low since the distribution of the main variable (VAS pain) in a funnel plot did not show asymmetries (Figure 3).

### 3.3. Quantitative Summary: Effects of High-Intensity Laser Therapy (HILT)

Effect on Pain

**Figure 4:**
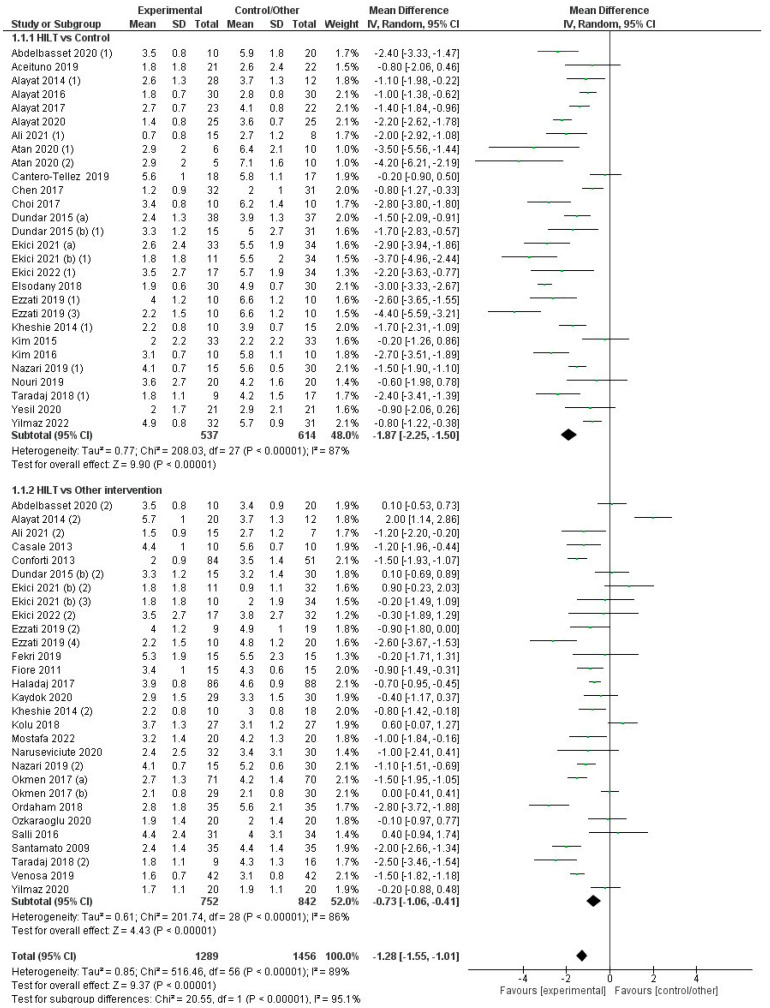
Forest plot for the overall effect on the pain VAS comparing high-intensity laser therapy (HILT) vs. control/another intervention and subgroup analysis depending on the comparator (HILT vs. control/HILT vs. another therapy). For studies with three arms: (1) Comparator: control or sham control. (2) Comparator: another therapy.

Figure 4 summarizes trials that assessed the effect of the interventions on pain measured on a VAS. Overall, the effectiveness of HILT for relieving pain was superior compared to control/sham groups and other conservative interventions (MD = −1.3 cm; CI95%: −1.6 to −1.0) with a high level of heterogeneity (I2 = 89%, *p* < 0.001). The quality of evidence for this outcome according to GRADE was low in terms of factors to rating down (serious risk of bias and serious inconsistency or heterogeneity). In the analysis by subgroups, the difference in the effectiveness of HILT versus control/sham (MD = −1.9 cm; CI95%: −2.3 to −1.5) was significantly superior (χ2 = 20.6, *p* < 0.001) than in the comparison of HILT against other conservative treatments (MD = −0.7 cm; CI95%: −1.1 to −0.4). However, the heterogeneity was high for both subgroups (I2 = 87% and 86%, respectively; *p* < 0.001). The effect of HILT on pain was superior to that observed in the control or sham stimulation groups for 23 of the 28 controlled trials [13,18,19,21,22,23,24,25,26,27,28,34,36,37,41,47,51,55,56,57,60], and also when compared with other conservative treatments in 15 of the 29 studies using this comparator [17,20,28,30,31,34,39,41,45,50,52,57]. On the contrary, only one trial [25] with high risk of bias reported a superior effect of one conservative treatment (exercising protocol) versus HILT in people with chronic lumbar pain (Figure 4). The effect of HILT was superior to ultrasound therapy (MD = −1.0 cm; CI95%: −1.8 to −0.3) [26,30,44,50,57] TENS or TENS plus ultrasound therapy (MD = −0.6 cm; CI95%: −1.2 to −0.02) [17,27,38,41,46,52,54], shock wave therapy (MD = −1.0 cm; CI95%: −1.8 to −0.2) [39], and LLLT (MD = −1.2 cm; CI95%: −2.0 to −0.5) [13,28,29,33,34,40,45,51] (Supplementary Appendix S3). No differences were found between HILT and other therapies, such as exercising and vertebral traction (MD = −0.1 cm; CI95%: −1.6 to 1.4), bandages and orthosis (MD = 0.1 cm; CI95%: −0.4 to 0.6) [22,26,40], or invasive radiofrequency (MD = 0.0 cm; CI95%: −0.4 to 0.4) [43] (Supplementary Appendix S3).

When an analysis was performed depending on the follow-up period (post-immediate, 3–9 weeks, and 10–24 weeks), no differences were found between HILT and control/sham groups. However, the effect tended to decrease as the follow-up period increased (Table 1 and Supplementary Appendix S3). No differences were observed in terms of dosage when comparing the effect of four dosages (≤10 J/cm^2^, 10–50 J/cm^2^, 50–100 J/cm^2^, 100–300 J/cm^2^), but the effect tended to decrease with higher dosages (Table 1 and Supplementary Appendix S3). Two trials were excluded from this analysis for not specifying the delivered dose [16,35]. In the comparison between subgroups in terms of anatomical location of musculoskeletal pain, no differences in the effect on pain were observed, but the effect tended to be greater for temporomandibular joint and, conversely, the effect did not reach statistical significance for the foot (Table 1 and Supplementary Appendix S3). In only four studies was the physiotherapist applying the HILT blinded [16,42,47,56]. No differences were found between subgroups blinded/not-blinded on pain (Chi^2^ = 2.8; *p* = 0.10): blinded group (MD= −1.2; CI95 % −2.1 to −0.3) vs. not-blinded group (MD = −2.0; CI 95% −2.4 to −1.6). 

### 3.4. Effect on Functionality

**Figure 5:**
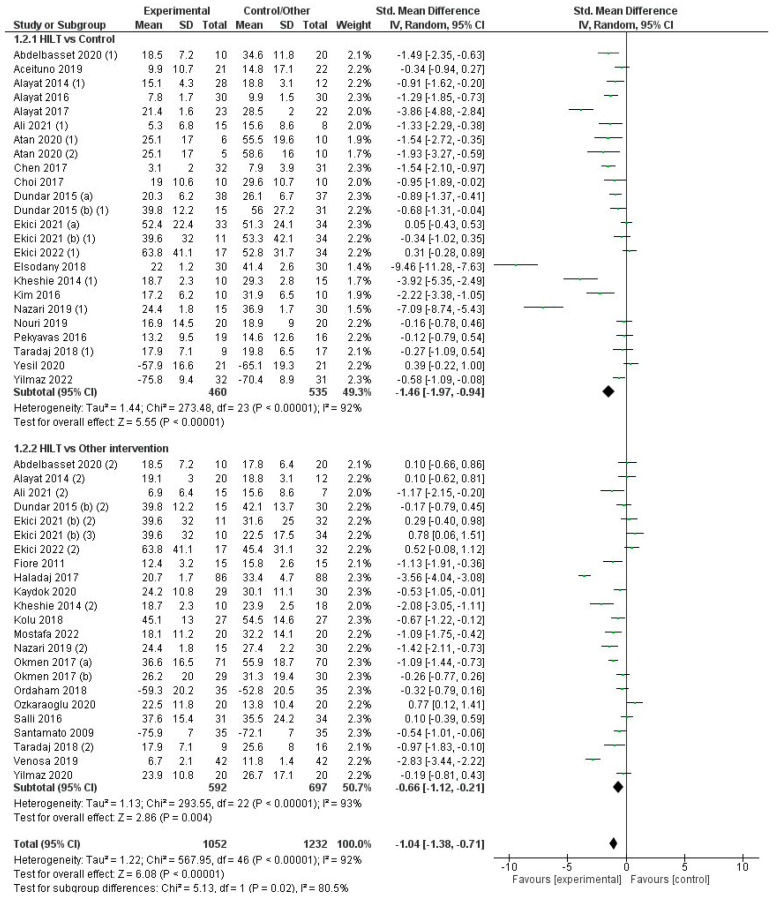
Forest plot for the overall effect on functionality comparing high-intensity laser therapy (HILT) vs. control/another intervention and subgroup analysis depending on the comparator (HILT vs. control/HILT vs. another therapy). For studies with three arms: (1) Comparator: control or sham control. (2) Comparator: another therapy.

When comparing HILT versus control/sham control groups and other conservative interventions, the overall effect on functionality was superior with HILT (SMD = −1.0; CI95%: −1.4 to −0.7) with a high level of heterogeneity (I2 = 92%; *p* < 0.001) (Figure 5). The quality of evidence for this outcome according to GRADE was moderate in terms of factors related to rating down (serious risk of bias and serious inconsistency or heterogeneity) and the factor related to rating up (magnitude of effect). In the analysis by subgroups, the difference between HILT and control/sham control (SMD = −1.5; CI95%: −2.0 to −0.9) was significantly greater (χ2 = 5.1; *p* = 0.02) than that observed when comparing HILT to other conservative treatments (SMD = −0.7; CI95%: −1.1 to −0.2). However, the level of heterogeneity was high (I2 = 92% and 93%, respectively; *p* < 0.001) in both subgroups (Figure 5). 

In 16 of the 24 controlled trials, the effect of HILT on functionality was superior to that reported in control or sham control groups [13,18,19,21,22,23,25,34,36,37,41,47,55,57,60]. The effect was also superior in 12 of the 23 studies that compared HILT versus other conservative treatments [30,31,33,34,38,39,41,44,50,51,52,57]. Only two trials by studies found a greater effect with other treatments. Ozkarauglu et al. and Ekici et al. reported a greater effect with TENS and occlusal splints in patients with low back pain and temporomandibular pain, respectively (Figure 5) [26,46]. The effect of HILT on functionality was superior to that of ultrasound therapy (SMD = −0.7; CI95%: −1.2 to −0.2) [26,30,44,50,57], shock wave therapy (SMD = −1.1; CI95%: −1.8 to −0.4) [39], and LLLT (SMD = −0.7; CI95%: −1.4 to −0.1) [13,33,34,45,51] (Supplementary Appendix S3). No differences were found between HILT and other treatments like TENS or TENS plus ultrasound therapy (SMD = −0.7; CI95%: −1.7 to 0.4) [27,38,41,46,52,54], exercising and vertebral tractions (SMD = −1.7; CI95%: −5.3 to 1.9) [25,31], bandages and orthosis (SMD = 0.2; CI95%: −0.3 to 0.7) [22,26,49], or invasive radiofrequency (SMD = −0.3; CI95%: −0.8 to 0.3) [43] (Supplementary Appendix S3).

No significant differences were found between HILT and control/sham control groups depending on the follow-up period (post-immediate, 3–9 weeks, and 10–24 weeks), but the effect tended to be greater in the long term (Table 1 and Supplementary Appendix S3). When four dosages were compared (≤10 J/cm^2^, 10–50 J/cm^2^, 50–100 J/cm^2^, 100–300 J/cm^2^), the greatest effect on functionality was achieved with 10–50 J/cm^2^, whereas 50–100 J/cm^2^ did not produce a significant effect. However, differences between these four subgroups did not reach statistical significance (Table 1 and Supplementary Appendix S3). Significant differences (*p* < 0.001) were observed in the comparison by subgroups in terms of the anatomical location of musculoskeletal pain, where the greatest effect on functionality was recorded at the knee and shoulder, and with no changes in functionality at the foot and temporomandibular pain (Table 1 and Supplementary Appendix S3). 

In only two studies was the physiotherapist applying the HILT blinded [42,47]. No differences were found between subgroups blinded/not-blinded on functionality (Chi^2^ = 0.1; *p* = 0.76): blinded group (SMD = −2.0; CI 95% −5.6 to 1.6) vs. not-blinded group (SMD = −1.4; CI 95% −1.9 to −0.9).

### 3.5. Effect on Secondary Variables: Range of Motion (ROM), Strength, and Quality of Life

**Figure 6:**
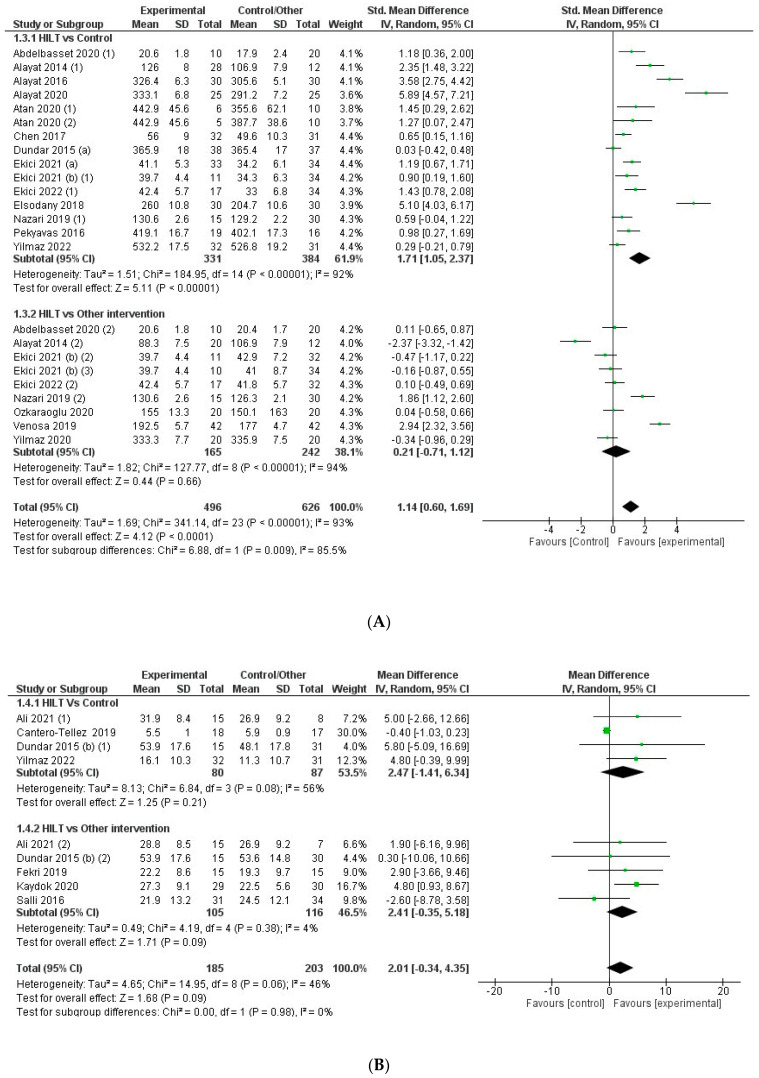
(**A**) Forest plot for the overall effect on range of motion (ROM) comparing high-intensity laser therapy (HILT) vs. control/another intervention and subgroup analysis depending on the comparator (HILT vs. control/HILT vs. another therapy); (**B**) forest plot for the overall effect on strength comparing high-intensity laser therapy (HILT) vs. control/another intervention and subgroup analysis depending on the comparator (HILT vs. control/HILT vs. another therapy). For studies with three arms: (1) Comparator: control or sham control. (2) Comparator: another therapy.

Overall, HILT was effective in improving the ROM (SMD = 1.1; CI95%: 0.6 to 1.7). The quality of evidence for the ROM variable according to GRADE was moderate in terms of factors related to rating down (serious risk of bias and serious inconsistency or heterogeneity) and the factor related to rating up (magnitude of effect). A significant difference in the ROM outcome was observed in the subgroup analysis depending on the comparator (control/other treatments) (χ2 = 6,9; *p* < 0.01). When comparing HILT versus control, an increased in ROM outcome was observed in favor of HILT (SMD = 1.7; CI95%: 1.1 to 2.4), but no differences were found when comparing with other treatments (SMD = 0.2; CI95%: −0.7 to 1.1) (Figure 6A). 

No significant differences were recorded in the overall effect on muscle strength (MD = 2.0 kg; CI95%: −0.3 to 4.4) or in the analysis by subgroups depending on the comparator (control/other treatments) (Figure 6B). 

**Figure 7:**
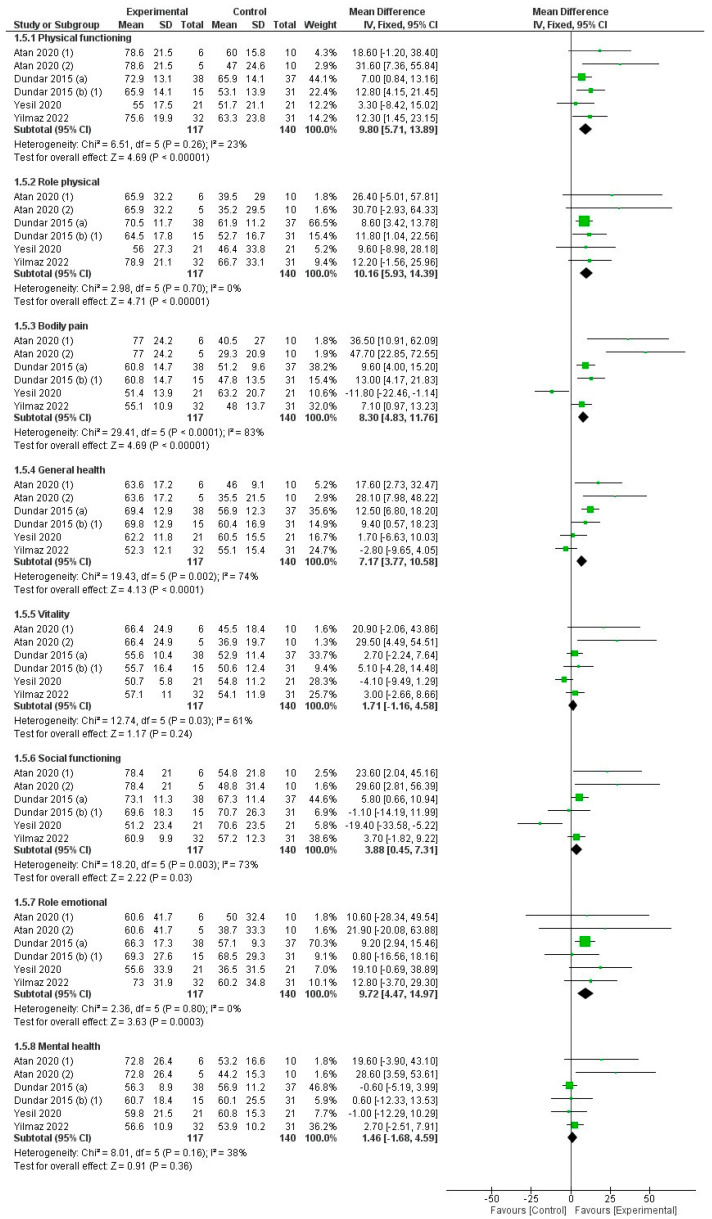
Forest plot for the overall effect on quality-of-life assessment by SF-36 questionary comparing high-intensity laser therapy (HILT) vs. control.

The quality of evidence for the strength variable according to GRADE was moderate in terms of the factor related to rating down (serious risk of bias). HILT was more effective for improving quality of life than control, especially in the four items of the SF-36 questionnaire related to physical health (physical functioning, role physical, bodily pain, and general health) and in two items related to mental health (role of emotional and social functioning), with no differences in the two remaining items related to mental health (vitality and mental health) (Figure 7). The quality of evidence for the quality-of-life variable according to GRADE was moderate in terms of the factor related to rating down (serious risk of bias). 

Of note, the previously registered protocol of this meta-analysis included a pooled quantitative analysis of adverse effects that in the end could not be conducted because only one adverse event was reported, consisting of allergy to HILT [60], and no adverse events were noted in control groups or others receiving conservative treatments.

## 4. Discussion

This meta-analysis showed that HILT is an effective treatment for improving pain and functionality in musculoskeletal disorders with low and moderate recommendation levels according to GRADE, respectively. The improvement in these variables was greater when comparing HILT versus control or sham than versus other conservative treatments. Some studies determined that a change of 1.4–2.0 cm on a musculoskeletal pain VAS can be considered clinically significant [61,62]. The difference of 1.9 cm on the pain VAS observed between HILT and control treatments can be considered clinically relevant, unlike the 0.7 cm difference observed between HILT and other conservative therapies. The standardized mean difference (SMD) is used to measure the magnitude of the effect, and an SMD of ≥0.8 is considered to represent a large effect [63]. The magnitude of the effect on functionality observed between HILT and control treatments can be considered large (SMD = 1.5), unlike the effect observed between HILT and other conservative therapies (SMD = 0.7). However, the improvement in functionality was large using HILT when comparing to shock wave therapy. This could be since the dose used in shock wave therapy (0.05 mJ/mm^2^) used in the included study was below the recommended dose [64].

The effects on pain and functionality of HILT versus control (MD = 1.9 cm on the pain VAS and SMD = 1.5 on functionality) observed in this meta-analysis were greater than those in a former meta-analysis by Song et al. [8] for musculoskeletal pain, which reported a pain reduction of MD= 1.0 cm on the VAS and SMD = 1.0 for the effect of HILT on functionality. Additionally, Song et al. [8] did not find differences in functionality when contrasting HILT against other conservative treatments. Of note, the number of included RCTs (n = 44) in the present meta-analysis was substantially greater than the RCTs (n = 12) in the meta-analysis by Song et al. [8].

In the analysis by subgroups for the follow-up period, an opposite tendency in the effect of the treatment was observed between the main variables (pain and functionality) when comparing HILT versus control. The effect of HILT on functionality showed a tendency to improve over time, whereas the improvement in pain tended to decrease, although statistical difference was not reached. These results are in agreement with the former meta-analysis by Song et al. [8] and another meta-analysis that assessed the effect of HILT on knee osteoarthritis [65].

In the analysis by subgroups to account for the dosage, lower doses (≤10 J/cm^2^) tended to achieve greater analgesic effects, although statistical difference was not reached. A study by Ezzati et al. [28] in patients with carpal tunnel syndrome also observed this effect, where after delivering two doses of HILT (8 J/cm^2^ versus 20 J/cm^2^), the analgesic effect of HILT was greater with 8 J/cm^2^. Along these lines, a preclinical study determined that a dose of 8 J/cm^2^ yielded the greatest antinociception, possibly due to the activation of the endogenous opioid system [66]. However, medium doses (>10 J/cm^2^ to 50 J/cm^2^) tended to achieve greater effect on functionality. Additionally, the present meta-analysis did not observe significant effects on functionality employing high doses of 50 and 100 J/cm^2^. Nevertheless, more comparative trials delivering diverse dosages of HILT are necessary to determine the potential influence of this parameter on the therapy effectiveness. 

In the analysis by subgroups depending on the location of pain, effect on pain by location was close to reaching statistical significance, with a clinically significant improvement in pain (1.6 cm to 3 cm on the VAS) for all anatomical locations except for the foot [53]. Two former meta-analyses assessing the effectiveness of HILT for treating two specific musculoskeletal disorders, knee osteoarthritis [65] and spinal disorders [67], which included six and nine RCTs, respectively, reported a lesser effect on pain reduction than the present meta-analysis. In terms of the effect on functionality, this meta-analysis found significant differences depending on the location, with the greatest effect observed at the knee and shoulder and without effect in temporomandibular and foot locations. Song et al. [8] reported the greatest effect at the neck and lower back, although no trial about the knee was included. The functionality results of this meta-analysis were very similar to those obtained by Alayat et al. in a previous meta-analysis about spinal disorders [67] and inferior to those observed in a former meta-analysis about knee osteoarthritis [65].

HILT could be considered a safe technique due to the absence of adverse effects reported by the authors and the similar number of abandonments in the experimental and control groups. However, 62.5% of the included trials did not specify these data. The previously published systematic reviews and meta-analyses [8,65,67] on HILT did not assess complications or adverse effects. The reported adverse effects of LLLT do not differ from those stated by patients exposed to placebo devices in trials [5]. However, the higher power that HILT employs makes it necessary to conduct further research where adverse effects or complications are systematically assessed. The analysis of secondary variables showed the effectiveness of HILT for improving both the ROM and quality of life with a “moderate” recommendation level according to GRADE, in contrast to muscle strength, which did not improve with the intervention and had a “moderate” recommendation level according to GRADE. To our knowledge, this is the first meta-analysis that assessed the effect of HILT on these variables.

### Study Limitations

An important limitation of this review is the large heterogeneity in the results of the assessed variables, which lowered the certainty of evidence. Strength and some domains of quality of life as assessed via the SF-36 were the only variables with moderate or low heterogeneity. The previous meta-analysis by Song et al. [8] also reported this high level of heterogeneity. Additionally, the analysis by subgroups did not reduce heterogeneity. Although determining the factors accounting for this high heterogeneity or inconsistency of the results was not possible, it could stem from the large variability in demographic characteristics of the samples, with ages ranging from 28 to 64 years, as well as from their clinical characteristics, such as the diverse pathologies that included etiology, duration, and severity of symptoms. Additionally, protocols for applying HILT were also heterogeneous. Another important limitation was the high risk of bias of the included trials, mainly regarding the blinding of participants and the researcher delivering the interventions. Hence, these results must be regarded with caution since the effect of HILT could be overestimated due to the large placebo effect that high-technology medical devices can produce [68].

## 5. Conclusions

This updated meta-analysis supports the effectiveness of HILT in improving pain, functionality, ROM, and quality of life in patients with musculoskeletal pain, without side effects. However, the certainty of this evidence was classified as “low” and “moderate”. The effectiveness of HILT varied depending on the location of the musculoskeletal disorder, and the greatest effect on pain was observed at the shoulder and knee. Future clinical research and reviews should be designed with a lower risk of bias in order to improve the certainty of this evidence.

## Figures and Tables

**Table 1:** Subgroup analysis of high-intensity laser therapy (HILT) versus control for the treatment of musculoskeletal pain, as measured on the pain VAS and functionality.

Outcome	Subgroup	Studies or Arms, n	Participants, n	Random Effect MD ^†^/SMD ^†^ (CI 95%)	Heterogeneity I^2^ % (*p* Value)	Difference between Subgroups Chi^2^ (*p* Value)
**VAS**	Follow-up period					
	Post-immediate	8	266	−2.1 (−2.7 to −1.5)	79% (*p* < 0.001)	1.0 (*p* = 0.59)
	3–9 weeks	15	807	−1.9 (−2.3 to −1.4)	87% (*p* < 0.001)	
	10–24 weeks	5	213	−1.5 (−2.8 to −0.1)	94% (*p* < 0.001)	
	Dosage					
	≤ 10 J/cm^2^	13	649	−2.2 (−2.7 to −1.6)	86% (*p* < 0.001)	0.82 (*p* = 0.85)
	10–50 J/cm^2^	4	194	−2.0 (−3.1 to −0.9)	96% (*p* < 0.001)	
	50–100 J/ cm^2^	2	95	−1.9 (−2.7 to −1.0)	72% (*p* = 0.06) ^a^	
	100–300 J/ cm^2^	7	247	−1.7 (−2.6 to −0.9)	73% (*p* = 0.002)	
	Location					
	Shoulder	6	263	−1.9 (−3.2 to −0.7)	94% (*p* < 0.001)	10.9 (*p* = 0.09)
	Elbow, wrist, thumb	5	185	−2.2 (−3.5 to −0.8)	92% (*p* < 0.001)	
	Neck	3	185	−1.6 (−2.3 to −0.8)	89% (*p* < 0.001)	
	Temporomandibular	3	201	−3.0 (−3.8 to −2.1)	53% (*p* = 0.12) ^a^	
	Low back	5	210	−1.8 (−2.7 to −1.0)	84% (*p* < 0.001)	
	Knee	5	200	−1.6 (−2.0 to −1.2)	62% (*p* = 0.03)	
	Foot	1	42	−0.9 (−2.1 to 0.3) ^b^	NA ^†^	
**FUNCTIONALITY**	Follow-up period					
	Post-immediate	8	251	−1.5 (−2.2 to −0.9)	80% (*p* < 0.001)	0.85 (*p* = 0.66)
	3–9 weeks	12	682	−1.3 (−2.0 to −0.6)	94% (*p* < 0.001)	
	10–24 weeks	4	178	−2.3 (−4.4 to −0.2)	97% (*p* < 0.001)	
	Dosage					
	≤10 J/cm^2^	12	604	−0.7 (−1.2 to −0.3)	85% (*p* < 0.001)	6.1 (*p* = 0.11)
	10–50 J/cm^2^	3	165	−4.8 (−8.5 to −1.0)	98% (*p* < 0.001)	
	50–100 J/ cm^2^	2	95	−3.6 (−10.3 to 3.0) ^b^	99% (*p* < 0.001)	
	100–300 J/ cm^2^	7	247	−1.1 (−1.7 to −0.6)	71% (*p* = 0.002)	
	Location					
	Shoulder	6	232	−2.1 (−3.6 to −0.6)	95% (*p* < 0.001)	40.1 (*p* < 0.001) **
	Elbow, wrist, thumb	2	91	−0.9 (−1.4 to −0.4)	19% (*p* = 0.27) ^a^	
	Neck	2	135	−1.1 (−1.5 to −0.7)	14% (*p* = 0.28) ^a^	
	Temporomandibular	3	201	0.0 (−0.4 to 0.4) ^b^	45% (*p* = 0.16) ^a^	
	Low back	5	210	−1.1 (−1.6 to −0.6)	62% (*p* = 0.03)	
	Knee	5	200	−3.4 (−5.8 to −1.1)	96% (*p* < 0.001)	
	Foot	1	42	0.4 (−0.2 to 1.0) ^b^	NA^†^	

(**) Bold values indicates a statistically significant **difference between subgroups** (*p* < 0.001). (**†**) MD: Mean difference for pain VAS. SMD: standardized mean difference for functionality. NA: Not applicable. (a) Heterogeneity did not reach statistical significance (*p* > 0.05). (b) Differences between HILT and control group did no reach statistical significance (*p* > 0.05).

## Data Availability

The data presented in this study are available on reasonable request from the corresponding author.

## Supplementary Materials

The following supporting information can be downloaded at: https://www.mdpi.com/article/10.3390/jcm12041479/s1, Supplementary Appendix S1: Search strategy, Supplementary Appendix S2: Summary of included studies, Supplementary Appendix S3: Risk of bias and forest plots of subgroups analysis.
