# Supplementary material for: High-Intensity Laser Therapy for Musculoskeletal Disorders: A Systematic Review and Meta-Analysis of Randomized Clinical Trials
Source: J Clin Med. 2023 Feb 13;12(4):1479. doi: 10.3390/jcm12041479 (PMC9963402; doi:10.3390/jcm12041479)
Supplement: Supplementary file 1 [file jcm-12-01479-s001.zip › Suplementary appendix S1.pdf]

Search Strategy

The following keywords were used for searching databases: *High-intensity laser therapy, High-power laser therapy, High intensity laser therapy, High power laser therapy, High level laser therapy, HILT, Pain, Pain (MESH), Function, Functionality, Quality of life, QoL,* which were combined as follows:

(High-intensity laser therapy OR High-power laser therapy OR High intensity laser therapy OR High power laser therapy OR HILT OR High level laser therapy) AND pain AND (Function OR Functionality OR Quality of life OR QoL).

("High-intensity laser therapy" OR "High-power laser therapy" OR "High intensity laser therapy" OR "High power laser therapy" OR "HILT" OR "High level laser therapy") AND "pain" AND ("Function" OR "Functionality" OR "Quality of life" OR "QoL").
