# Supplementary material for: High-Intensity Laser Therapy for Musculoskeletal Disorders: A Systematic Review and Meta-Analysis of Randomized Clinical Trials
Source: J Clin Med. 2023 Feb 13;12(4):1479. doi: 10.3390/jcm12041479 (PMC9963402; doi:10.3390/jcm12041479)
Supplement: Supplementary file 1 [file jcm-12-01479-s001.zip › Suplementary appendix S2.pdf]

Supplementary appendix 2: Summary of included studies

First Author / Year / Country	Musculoskeletal disorder / Size randomized (n) analyzed [n]	Intervention arms. Randomized (n) analyzed [n]	HILT parameters: Laser type/ wavelength (nm)/ power (W)/ energy density (J/cm2)/ number of sessions/ sessions per week	Time points / Outcome measures	Losses to follow-up / Adverse events
Abdelbasset 2020 Saudi Arabia	Chronic low back pain (30) [30]	HILT+EX (20) [20]<br>LLLT+EX (20) [20]<br>EX (20) [20]	GADL / 1064 / 12 / 150 / 24 / 2	Post-immediate /<br>VAS, ODI, EQ-5D-3L, ROM	None / NR
Aceituno 2019 Spain	SAIS (46) [43]	HILT+EX (23) [21]<br>Sham HILT + EX (23) [22]	Nd:YAG / 1064 / 15 / 300 / 15 / 5	Post-immediate; FU 4 and 12 w /<br>VAS, SPADI, CMS, Q-DASH, PPT	3 / None
Alayat 2014 Saudi Arabia	Chronic low back pain (72) [72]	HILT + EX (28) [28]<br>Sham HILT + EX (24) [24]<br>HILT (20) [20]	Nd:YAG / 1064 / 10.5 / 4.87 / 12 / 3	Post-immediate; FU 8 w /<br>VAS, ODI, RMDQ, ROM	None / NR
Alayat 2016 Saudi Arabia	Chronic neck pain (60) [60]	HILT + EX (30) [30]<br>Sham HILT + EX (30) [30]	Nd:YAG / 1064 / 10.5 / 27.3 / 12 / 2	Post-immediate /<br>VAS, NDI, ROM	None / NR
Alayat 2017 Saudi Arabia	Knee osteoarthritis (67) [67]	HILT + Med + EX (23) [23]<br>Med + EX (22) [22]<br>Sham HILT + EX (22) [22]	Nd:YAG / 1064 / 10.5 / 15 / 12 / 2	Post-immediate; FU 6 w /<br>VAS, WOMAC, US	None / NR
Alayat 2020 Saudi Arabia	Cervical MPS (50) [50]	HILT + PPRT + EX (25) [25]<br>Sham HILT + PPRT + EX (25) [25]	Nd:YAG / 1064 / 10.5 / 2.64 / 12 / 3	Post-immediate /<br>VAS, PPT, ROM	NR / NR
Ali 2021 Egypt	Lateral epicondylitis (45) [45]	HILT+US (15) [15]<br>HILT (15) [15]<br>US (15) [15]	NR / 808-915 / 3.2 / 106 / 12 / 2	Post-immediate /<br>VAS, DASH, strength	None / NR
Akaltun 2021 Turkey	Knee osteoarthritis (40) [40]	HILT+EX (20) [20]<br>Sham HILT+EX (20) [20]	Nd:YAG / 1064 / 12 / 12-120 / 10 / 5	Post-immediate; FU 4 w /<br>VAS, WOMAC, ROM, sonography	None / NR
Angelova 2016 Bulgaria	Knee osteoarthritis (72) [72]	HILT (37) [37]<br>Sham HILT (35) [37]	Semiconductive neodymium laser IV / 1064 / 12 / 12 – 120 / 7 / NR	Post-immediate; FU 4 and 12 w /<br>VAS, dolorimetry, pedobaryc analysis	None / NR

Supplementary appendix 2: Summary of included studies

Atan<br>2020<br>Turkey	Adhesive capsulitis<br>(36) [31]	HILT + EX (12) [11]<br>Sham HILT + EX (12) [10]<br>EX (12) [10]	Nd:YAG / 1064 / 15 / 300 /<br>15 / 5	Post-immediate /<br>VAS, ROM, SPADI, SF-36	5 / 1
Boyraz<br>2015<br>Turkey	Lumbar disc herniation<br>(65) [65]	HILT + EX (20) [20]<br>US + EX (25) [25]<br>Med + EX (20) [20]	GAAL / 1064 / 3.8 / NR /<br>10 / 5	Post-immediate; FU 12 w /<br>VAS, ODI, SF-36	None / NR
Cantero-<br>Téllez<br>2019<br>Spain	Thumb Carpometacarpal<br>Osteoarthritis (43) [43]	HILT (22) [22]<br>Sham HILT (21) [21]	Class IV laser / 800 + 970 /<br>1.5 / NR / 12 / 3	FU 4 and 12 w /<br>VAS, strength	None / NR
Casale<br>2013<br>Italy	Carpal tunnel syndrome<br>(20) [20]	HILT (10) [10]<br>TENS (10) [10]	NR / 830 – 1064 / 25 / 250<br>/ 15 / 5	Post-immediate /<br>VAS, Neurophysiological parameters	None / NR
Chen<br>2017<br>China	Lumbar disc protrusion<br>(63) [63]	HILT + Spinal traction (32) [32]<br>Spinal traction (31) [32]	GADL / 1064 / 12 / 150 / 10<br>/ 5	FU 2 and 6 w /<br>VAS, ODI, ROM, SLRT	None / NR
Choi<br>2017<br>Korea	Chronic back pain<br>(20) [20]	HILT + CPT (10) [10]<br>CPT (10) [10]	Nd:YAG / 1064 / NR / 1.37<br>/ 12 / 3	Post-immediate /<br>VAS, ODI	None / NR
Conforti<br>2013<br>Italy	Whiplash<br>(135) [135]	HILT (84) [84]<br>CPT (51) [51]	NR / 780 – 1100 / 12 /<br>6.5 / 5 / 5	Post-immediate /<br>VAS, Return-to-work time	None / NR
Dundar<br>2015<br>Turkey	Trapezius MPS<br>(76) [75]	HILT + EX (38) [38]<br>Sham HILT + EX (38) [37]	Nd:YAG / 1064 / 10.5 /<br>3.17 / 15 / 5	Post-immediate; FU 8 w /<br>VAS, NDI, ROM, SF-36	1 / None
Dundar<br>2015<br>Turkey	Lateral epicondylitis<br>(93) [91]	HILT (31) [30]<br>Sham HILT (31)[31]<br>Brace (31) [30]	Nd:YAG / 1064 / 10.5 /<br>6.06 / 15 / 5	Post-immediate; FU 8 w /<br>VAS, PRTEE, SF-36, strength,<br>sonography	2 / None
Elsodany<br>2018<br>Saudi Arabia	SAIS<br>(60) [60]	HILT + EX (30) [30]<br>Sham HILT + EX (30) [30]	Nd:YAG / 1064 / 10.5 / 40 /<br>12 / 3	Post-immediate; FU 12 and 24 w /<br>VAS, SPADI, ROM	None / NR
Ekici<br>2021 (a)	Myogenic TMJD<br>(70) [67]	HILT (35) [33]<br>Sham HILT (35) [34]	Pulsed Nd:YAG / 1064 /<br>10.5 / 0.36-0.61 / 15 / 5	Post-immediate; FU 8 w /<br>VAS, JFLS-20, OHIP-14, ROM	3 / None

Supplementary appendix 2: Summary of included studies

Turkey					
Ekici (2021) (b) Turkey	Discal TMJD (140) [132]	Occlusal splint (35) [34]<br>US (35) [32]<br>HILT (35) [32]<br>EX (35) [34]	Pulsed Nd:YAG / 1064 / 10.5 / 0.36-0.61 / 20 / 4	Post-immediate; FU 8 w / VAS, JFLS-20, OHIP-14, ROM	8 / None
Ekici 2022 Turkey	TMJ (102) [100]	HILT+EX (34) [34]<br>TENS+EX (34) [34]<br>EX (34) [34]	Pulsed Nd:YAG / 1064 / 10.5 / 0.36-0.61 / 15 / 5	Post-immediate; FU 8 w / VAS, JFLS-20, OHIP-14, ROM	2 / None
Ezzati 2019 Iran	Carpal tunnel syndrome (100) [98]	HILT-LF + EX (20) [20]<br>HILT-HF + EX (20) [19]<br>LLLT-LF + EX (20) [20]<br>LLLT-HF + EX (20) [19]<br>EX (20) [20]	NR / 808 / 1.6 / 8 – 20 / 5 / NR	FU 3 weeks / VAS, Neurophysiological parameters	2 / NR
Fekri 2019 Iran	Lateral epicondylitis (30) [30]	HILT + TENS + US + EX (15) [15]<br>LLLT + TENS + US + EX (15) [15]	NR / 808 / 3.3 / 13.89 / 10 / 6	Post-immediate / VAS, PPT, strength	None / NR
Fiore 2011 Italy	Low back pain (30) [30]	HILT (15) [15]<br>US (15) [15]	NR / 1064 / 6 / 0.71 / 15 / 5	Post-immediate / VAS, ODI	None / NR
Haladaj 2017 Poland	Cervical spondylosis (174) [174]	HILT (86) [86]<br>Cervical traction (88) [88]	NR / 980 / 7 / 55 / 10 / 5	Post-immediate; FU 4 and 12 w / VAS, NDI	None / NR
Hojjati 2020 Iran	Carpal tunnel syndrome (45)[45]	HILT + Wrist splint (15) [15]<br>LLLT + Wrist splint (15) [15]<br>Wrist splint (15) [15]	NR / 1064/ 5/ 20/ NR/NR	Post-immediate; FU 12 w / VAS, BQ-SS, Neurophysiological parameters, strength	None / NR
Kaydok 2020 Turkey	Lateral epicondylitis (60) [59]	HILT + Brace (30)[29]<br>LLLT + Brace (30)[30]	Nd: YAG / 1064 / 8 / 6-150 / 9 / 3	Post-immediate / VAS, Q-DASH, SF-36, strength	1 / None
Kheshie 2014 Saudi Arabia	Knee osteoarthritis (60) [53]	HILT + EX (20)<br>LLLT + EX (18)<br>Sham HILT + EX (15)	Nd: YAG / 1064 / 10.5 / 3.65 / 12 / 2	Post-immediate / VAS, WOMAC	5 / NR

Supplementary appendix 2: Summary of included studies

Kim 2015 Korea	Frozen shoulder (66) [66]	HILT + NSAIDs + EX (33)<br>Sham HILT + NSAIDs + EX (33)	Nd: YAG / 1064 / 30 / NR / 9 / 3	Post-immediate; FU 5 and 9 w / VAS, ROM	None / None
Kim 2016 Korea	Knee osteoarthritis (20) [20]	HILT + PT (10) [10]<br>CPT (10) [10]	Nd: YAG/ 1064 / NR / 1.5 / 12 / 3	FU 4 w / VAS, WOMAC	None / NR
Kolu 2018 Turkey	Chronic lumbar discopathy (54) [54]	HILT + hot pack + EX (27) [27]<br>TENS + US + hot pack + EX (27) [27]	Nd: YAG / 1064 / 10 / 132 / 10 / 5	Post-immediate; FU 4 w / VAS, ODI	None / NR
Mostafa 2022 Egypt	Knee osteoarthritis (40) [40]	ESWT + CPT (20)[20]<br>HILT + CPT (20)[20]	Nd: YAG /1064/ 10.5/ 1.5/ 12 / 3	Post-immediate / VAS, WOMAC, 6MWT	None / NR
Naruseviciute 2020 Lithuania	Plantar fasciitis (109) [62]	HILT + pain education (54)[32]<br>LLLT+ pain education (55)[30]	Nd: YAG / 1064 / 7 / 120 / 8 / 3	Post-immediate; FU 4 w / VAS, PPT, sonography, medication	47 / None
Nazari 2019 Iran	Knee osteoarthritis (93) [90]	HILT + EX (31)[30]<br>TENS+ US + EX (31)[30]<br>EX (31)[30]	Nd: YAG / 1064 / 5 / 60 / 12 / 3	Post-immediate; FU 8 w / VAS, WOMAC, ROM, TUGT, 6MWT	3 / None
Nouri 2019 Iran	Patellofemoral pain syndrome (44) [40]	HILT + EX (22)[20]<br>Sham HILT + EX (22)[20]	Nd: YAG / 1064 / 10 / 120 / 5 / 3 – 2	FU 2 and 10 w / VAS, WOMAC, KSQ	4 / None
Ökmen 2017 Turkey	SAIS (70) [59]	HILT + EX (35)[29]<br>SNR + EX (35)[30]	NR / 1064 / 8 / 20 – 100 / 7 / 7	Post-immediate; FU 4, 12 and 24 w / VAS, SPADI, NHP	11 / None
Ökmen 2017 Turkey	SAIS (154) [141]	HILT + hot pack + TENS + balneotherapy + EX (77)[71]<br>US + hot pack + TENS + balneotherapy + EX (77)[70]	Nd:YAG / 1064 / 8 / 20 – 100 / 7 / 7	Post-immediate; FU 4 w / VAS, SPADI	13 / None
Ordahan 2018 Turkey	Plantar fasciitis (75) [70]	HILT + insole + EX (38)[35]<br>LLLT + insole + EX (37)[35]	Nd: YAG / 1064 / 8 / 6 – 150 / 9 / 3	Post-immediate / VAS, HTI, FAOS	5 / NR
Ozkaraoglu 2020	Lumbar disc herniation (40) [40]	HILT + US + hot pack + EX (20) [20]	Nd: YAG / 1064 / 12 / 10 / 20 / 5	FU 4 w / VAS, ODI, ROM, BDI	NR / NR

Supplementary appendix 2: Summary of included studies

Turkey		TENS + US + hot pack + EX (20) [20]			
Pekyavas 2016 Turkey	SAIS (70) [70]	HILT+MT+KT+EX (19) [19]<br>EX (15) [15]<br>KT + EX (20) [20]<br>MT + KT + EX (16) [16]	Nd: YAG / 1064 / 10.5 / 3.66 / 15 / 3	Post-immediate/ ROM, SPADI	None / NR
Salli 2016 Turkey	Lateral epicondylitis (65) [65]	HILT (31) [31]<br>Bandage (34) [34]	NR / NR / 6 / 6 – 150 / 10 / NR	FU 6 w / VAS, DASH, strength, SF-36	None / NR
Santamato 2009 Italy	SAIS (70) [70]	HILT (35) [35]<br>US (35) [35]	Nd: YAG / 1064 / 6 / 3.66 / 10 / 5	Post-immediate / VAS, CMS, SST	None / NR
Taradaj 2018 Poland	Lumbar degenerative disc disease (68) [68]	HILT (18) [18]<br>Sham HILT (17) [17]<br>LLLT (16) [16]<br>Sham LLLT (17) [17]	GAAL / 1064 / 10 / 60 / 15 / 5	Post-immediate; FU 4 and 12 w / VAS, ODI, RMDQ, LQIP, Lasegue test, Schober´s test	NR / NR
Venosa 2019 Italy	Cervical spondylosis (84) [84]	HILT + EX (42) [42]<br>TENS + US + EX (42) [42]	Nd: YAG / 1064 / 10.5 / 0.51 – 1.78 / 12 / NR	FU 4 w / VAS, NDI, ROM	NR / None
Yesil 2020 Turkey	Calcaneal spur (42) [42]	HILT + EX (21) [21]<br>Sham HILT + EX (21) [21]	Nd: YAG / 1064 / 10.5 / 8.26 / 15 / 5	Post-immediate; FU 8 w / VAS, RMS, SF-36, FAOS, pedobaryc analysis	None / None
Yilmaz 2020 Turkey	Cervical disc herniation (46) [40]	HILT + EX (23)[20]<br>US + TENS + EX (23)[20]	Nd: YAG / 1064 / 8 / 5 / 20 / 5	Post-immediate / VAS, NDI, ROM	6 / None
Yilmaz 2022 Turkey	SAIS (63) [63]	HILT + EX (32) [32]<br>Sham HILT + EX (31) [31]	Pulsed Nd:YAG / 1064 / 10.5 / 0.36 – 1.07 / 15 / 5	Post-immediate; FU 12 w / VAS, CMS, SF-36, ROM, strength	None / None

Nm: nanometers; W: watts; HILT: high intensity laser therapy; LLLT: low level laser therapy; EX: exercise; GADL: gallium-arsenide diode laser; VAS: visual aalogic scale; ODI: Oswestry Disability Index; EQ-5D-3L: EuroQoL-5 Dimensions-3 Levels; ROM: range of motion; NR: not reported; SAIS: subacromial impingement syndrome; Nd:YAG: neodymium-doped yttrium aluminium garnet; FU: follow-up; w: weeks; SPADI: Shoulder Pain and Disability Index; CMS: Constant-Murley Score; Q-DASH: Quick Disabilities of the arm, shoulder and hand questionnaire; PPT: Pressure Pain Threshold; RMDQ: Roland-Morris Disability Questionnaire; NDI = Neck and disability index; Med: medication; WOMAC = Western Ontario and McMaster Universities

Supplementary appendix 2: Summary of included studies

Osteoarthritis Index; US: ultrasound; MPS: myofascial pain syndrome; PPRT = Progressive pressure release technique; SF-36: Short Form 36 Health Survey Questionnaire; GAAL = Gallium Aluminium Arsenide Laser; TENS: Transcutaneous Electrical Nerve Stimulation; SLRT: Straight leg raising test; CPT: Conventional physical therapy; PRTEE: Patient-Rated Tennis Elbow Evaluation; TMJD: Temporomandibular joint dysfunction; JFLS-20: Jaw Functional Limitation Scale-20; OHIP-14: Oral Health Impact Profile-14; LF: Low frequency; HF: High frequency; BQ-SS = Boston Symptom Severity Scale; NSAIDs: Non-steroidal anti-inflammatory drugs; ESWT = Extracorporeal shockwave therapy; 6MWT = 6-min walk test; TUGT = Time Up and Go test; KSQ = Kujala Score Questionnaire; SNR = Suprascapular nerve radiofrequency; NHP: Nottingham Health Profile; HTI: Heel Tenderness Index; FAOS: *Foot* and Ankle Outcome Score; BDI: Beck Depression Inventory; MT: manual therapy; KT: kinesiotaping; SST: Simple Shoulder Test; LQIP: Laitinen Questionnaire Indicators of Pain; RMS: Roles and Maudsley Score.
