# Supplementary material for: High-Intensity Laser Therapy for Musculoskeletal Disorders: A Systematic Review and Meta-Analysis of Randomized Clinical Trials
Source: J Clin Med. 2023 Feb 13;12(4):1479. doi: 10.3390/jcm12041479 (PMC9963402; doi:10.3390/jcm12041479)

**Risk of bias summary: review authors' judgements about each risk-of-bias item for each included study**

| | Random sequence generation (selection bias) | Allocation concealment (selection bias) | Blinding of participants (performance bias) | Blinding of personnel (performance bias) | Blinding of outcome assessment (detection bias) | Incomplete outcome data (attrition bias) | Selective reporting (reporting bias) |
|---|---|---|---|---|---|---|---|
| Abdelbasset 2020 | + | ? | − | − | + | + | ? |
| Aceituno 2019 | − | − | + | − | + | + | + |
| Akaltun 2021 | + | ? | + | − | + | + | ? |
| Alayat 2014 | + | ? | + | − | − | + | ? |
| Alayat 2016 | + | ? | + | + | ? | + | ? |
| Alayat 2017 | + | ? | ? | − | + | + | ? |
| Alayat 2020 | + | ? | + | + | ? | + | ? |
| Ali 2021 | ? | ? | − | − | ? | + | ? |
| Angelova 2016 | − | − | + | − | − | ? | ? |
| Atan 2020 | + | + | ? | − | + | + | + |
| Boyraz 2015 | ? | − | − | − | − | ? | ? |
| Cantero-Tellez 2019 | + | + | + | + | + | + | + |
| Casale 2013 | + | ? | − | − | ? | + | ? |
| Chen 2017 | + | ? | − | − | ? | + | ? |
| Choi 2017 | ? | − | − | − | − | + | ? |
| Conforti 2013 | ? | ? | − | − | + | + | ? |
| Dundar 2015 (a) | + | + | + | − | + | + | ? |
| Dundar 2015 (b) | + | + | ? | − | + | + | ? |
| Ekici 2021 (a) | + | ? | + | − | + | + | ? |
| Ekici 2021 (b) | + | ? | − | − | + | + | ? |
| Ekici 2022 | + | + | − | − | + | + | ? |
| Elsodany 2018 | + | ? | + | − | + | + | ? |
| Ezzati 2019 | + | ? | ? | − | + | + | + |
| Fekri 2019 | ? | ? | ? | − | ? | + | ? |
| Fiore 2011 | + | + | − | − | + | + | ? |
| Haladaj 2017 | ? | ? | − | − | − | + | ? |
| Hojjati 2020 | ? | ? | − | − | + | + | + |
| Kaydok 2020 | + | + | ? | − | + | + | ? |
| Kheshie 2014 | + | ? | ? | − | − | + | ? |
| Kim 2015 | + | ? | + | − | + | + | ? |
| Kim 2016 | ? | ? | − | − | − | + | ? |
| Kolu 2018 | ? | ? | − | − | − | + | ? |
| Mostafa 2022 | + | + | − | − | + | + | + |
| Naruseviciute 2020 | + | + | ? | − | − | − | − |
| Nazari 2019 | + | + | − | − | + | + | + |
| Nouri 2019 | + | + | + | + | ? | + | + |
| Okmen 2017 (a) | + | ? | − | − | + | + | ? |
| Okmen 2017 (b) | + | ? | − | − | + | + | + |
| Ordaham 2018 | + | + | ? | − | + | + | ? |
| Ozkaraoglu 2020 | ? | ? | − | − | + | ? | ? |
| Pekyavas 2016 | + | ? | − | − | + | − | − |
| Salli 2016 | + | ? | − | − | − | + | ? |
| Santamato 2009 | + | + | − | − | + | + | ? |
| Taradaj 2018 | − | − | ? | − | + | ? | ? |
| Venosa 2019 | + | ? | − | − | + | ? | ? |
| Yesil 2020 | + | + | + | − | + | + | + |
| Yilmaz 2020 | + | ? | − | − | + | + | ? |
| Yilmaz 2022 | + | + | + | − | + | + | ? |

Forest plot for the effect on **VAS pain** comparing high-intensity laser therapy vs. other treatments. Subgroups analysis by treatment.

| Study or Subgroup | Experimental Mean | Experimental SD | Experimental Total | Other treatment Mean | Other treatment SD | Other treatment Total | Weight | Mean Difference IV, Random, 95% CI |
|---|---|---|---|---|---|---|---|---|
| **5.1.1 HILT vs. Ultrasound** | | | | | | | | |
| Ali 2021 (2) | 1.5 | 0.9 | 15 | 2.7 | 1.2 | 15 | 3.5% | -1.20 [-1.96, -0.44] |
| Ekici 2021 (b) (2) | 1.8 | 1.8 | 16 | 0.9 | 1.1 | 32 | 3.2% | 0.90 [-0.06, 1.86] |
| Fiore 2011 | 3.4 | 1 | 15 | 4.3 | 0.6 | 15 | 3.8% | -0.90 [-1.49, -0.31] |
| Okmen 2017 (a) | 2.7 | 1.3 | 71 | 4.2 | 1.4 | 70 | 4.0% | -1.50 [-1.95, -1.05] |
| Santamato 2009 | 2.4 | 1.4 | 35 | 4.4 | 1.4 | 35 | 3.7% | -2.00 [-2.66, -1.34] |
| **Subtotal (95% CI)** | | | **152** | | | **167** | **18.1%** | **-1.00 [-1.75, -0.25]** |
| Heterogeneity: Tau² = 0.61; Chi² = 26.78, df = 4 (P < 0.0001); I² = 85% | | | | | | | | |
| Test for overall effect: Z = 2.62 (P = 0.009) | | | | | | | | |
| **5.1.2 HILT vs. TENS / TENS+US** | | | | | | | | |
| Casale 2013 | 4.4 | 1 | 10 | 5.6 | 0.7 | 10 | 3.5% | -1.20 [-1.96, -0.44] |
| Ekici 2022 (2) | 3.5 | 2.7 | 34 | 3.8 | 2.7 | 32 | 2.6% | -0.30 [-1.60, 1.00] |
| Kolu 2018 | 3.7 | 1.3 | 27 | 3.1 | 1.2 | 27 | 3.6% | 0.60 [-0.07, 1.27] |
| Nazari 2019 (2) | 4.1 | 0.7 | 30 | 5.2 | 0.6 | 30 | 4.1% | -1.10 [-1.43, -0.77] |
| Ozkaraoglu 2020 | 1.9 | 1.4 | 20 | 2 | 1.4 | 20 | 3.3% | -0.10 [-0.97, 0.77] |
| Venosa 2019 | 1.6 | 0.7 | 42 | 3.1 | 0.8 | 42 | 4.1% | -1.50 [-1.82, -1.18] |
| Yilmaz 2020 | 1.7 | 1.1 | 20 | 1.9 | 1.1 | 20 | 3.6% | -0.20 [-0.88, 0.48] |
| **Subtotal (95% CI)** | | | **183** | | | **181** | **24.9%** | **-0.60 [-1.18, -0.02]** |
| Heterogeneity: Tau² = 0.48; Chi² = 42.43, df = 6 (P < 0.00001); I² = 86% | | | | | | | | |
| Test for overall effect: Z = 2.02 (P = 0.04) | | | | | | | | |
| **5.1.3 HILT vs. Shock waves** | | | | | | | | |
| Mostafa 2022 | 3.2 | 1.4 | 20 | 4.2 | 1.3 | 20 | 3.4% | -1.00 [-1.84, -0.16] |
| **Subtotal (95% CI)** | | | **20** | | | **20** | **3.4%** | **-1.00 [-1.84, -0.16]** |
| Heterogeneity: Not applicable | | | | | | | | |
| Test for overall effect: Z = 2.34 (P = 0.02) | | | | | | | | |
| **5.1.4 HILT vs. LLLT** | | | | | | | | |
| Abdelbasset 2020 (2) | 3.5 | 0.8 | 20 | 3.4 | 0.9 | 20 | 3.9% | 0.10 [-0.43, 0.63] |
| Ezzati 2019 (2) | 4 | 1.2 | 9 | 4.9 | 1 | 19 | 3.3% | -0.90 [-1.80, 0.00] |
| Ezzati 2019 (4) | 2.2 | 1.5 | 10 | 4.8 | 1.2 | 20 | 3.0% | -2.60 [-3.67, -1.53] |
| Fekri 2019 | 5.3 | 1.9 | 15 | 5.5 | 2.3 | 15 | 2.3% | -0.20 [-1.71, 1.31] |
| Kaydok 2020 | 2.9 | 1.5 | 29 | 3.3 | 1.5 | 30 | 3.5% | -0.40 [-1.17, 0.37] |
| Kheshie 2014 (2) | 2.2 | 0.8 | 20 | 3 | 0.8 | 18 | 3.9% | -0.80 [-1.31, -0.29] |
| Naruseviciute 2020 | 2.4 | 2.5 | 32 | 3.4 | 3.1 | 30 | 2.4% | -1.00 [-2.41, 0.41] |
| Ordaham 2018 | 2.8 | 1.8 | 35 | 5.6 | 2.1 | 35 | 3.2% | -2.80 [-3.72, -1.88] |
| Taradaj 2018 (2) | 1.8 | 1.1 | 18 | 4.3 | 1.3 | 16 | 3.4% | -2.50 [-3.31, -1.69] |
| **Subtotal (95% CI)** | | | **188** | | | **203** | **28.8%** | **-1.23 [-1.96, -0.49]** |
| Heterogeneity: Tau² = 1.03; Chi² = 57.18, df = 8 (P < 0.00001); I² = 86% | | | | | | | | |
| Test for overall effect: Z = 3.28 (P = 0.001) | | | | | | | | |
| **5.1.5 HILT vs. Exercises and tractions** | | | | | | | | |
| Alayat 2014 (2) | 5.7 | 1 | 20 | 3.7 | 1.3 | 24 | 3.6% | 2.00 [1.32, 2.68] |
| Conforti 2013 | 2 | 0.9 | 84 | 3.5 | 1.4 | 51 | 4.0% | -1.50 [-1.93, -1.07] |
| Haladaj 2017 | 3.9 | 0.8 | 86 | 4.6 | 0.9 | 88 | 4.2% | -0.70 [-0.95, -0.45] |
| **Subtotal (95% CI)** | | | **190** | | | **163** | **11.8%** | **-0.10 [-1.58, 1.38]** |
| Heterogeneity: Tau² = 1.66; Chi² = 73.48, df = 2 (P < 0.00001); I² = 97% | | | | | | | | |
| Test for overall effect: Z = 0.13 (P = 0.90) | | | | | | | | |
| **5.1.6 HILT vs. Bandages and orthosis** | | | | | | | | |
| Dundar 2015 (b) (2) | 3.3 | 1.2 | 30 | 3.2 | 1.4 | 30 | 3.7% | 0.10 [-0.56, 0.76] |
| Ekici 2021 (b) (3) | 1.8 | 1.8 | 16 | 2 | 1.9 | 34 | 2.9% | -0.20 [-1.29, 0.89] |
| Salli 2016 | 4.4 | 2.4 | 31 | 4 | 3.1 | 34 | 2.5% | 0.40 [-0.94, 1.74] |
| **Subtotal (95% CI)** | | | **77** | | | **98** | **9.1%** | **0.08 [-0.44, 0.60]** |
| Heterogeneity: Tau² = 0.00; Chi² = 0.48, df = 2 (P = 0.79); I² = 0% | | | | | | | | |
| Test for overall effect: Z = 0.29 (P = 0.77) | | | | | | | | |
| **5.1.9 HILT vs. Radiofrequency** | | | | | | | | |
| Okmen 2017 (b) | 2.1 | 0.8 | 29 | 2.1 | 0.8 | 30 | 4.0% | 0.00 [-0.41, 0.41] |
| **Subtotal (95% CI)** | | | **29** | | | **30** | **4.0%** | **0.00 [-0.41, 0.41]** |
| Heterogeneity: Not applicable | | | | | | | | |
| Test for overall effect: Z = 0.00 (P = 1.00) | | | | | | | | |
| **Total (95% CI)** | | | **839** | | | **862** | **100.0%** | **-0.72 [-1.05, -0.39]** |
| Heterogeneity: Tau² = 0.68; Chi² = 242.02, df = 28 (P < 0.00001); I² = 88% | | | | | | | | |
| Test for overall effect: Z = 4.24 (P < 0.0001) | | | | | | | | |
| Test for subgroup differences: Chi² = 16.85, df = 6 (P = 0.010), I² = 64.4% | | | | | | | | |

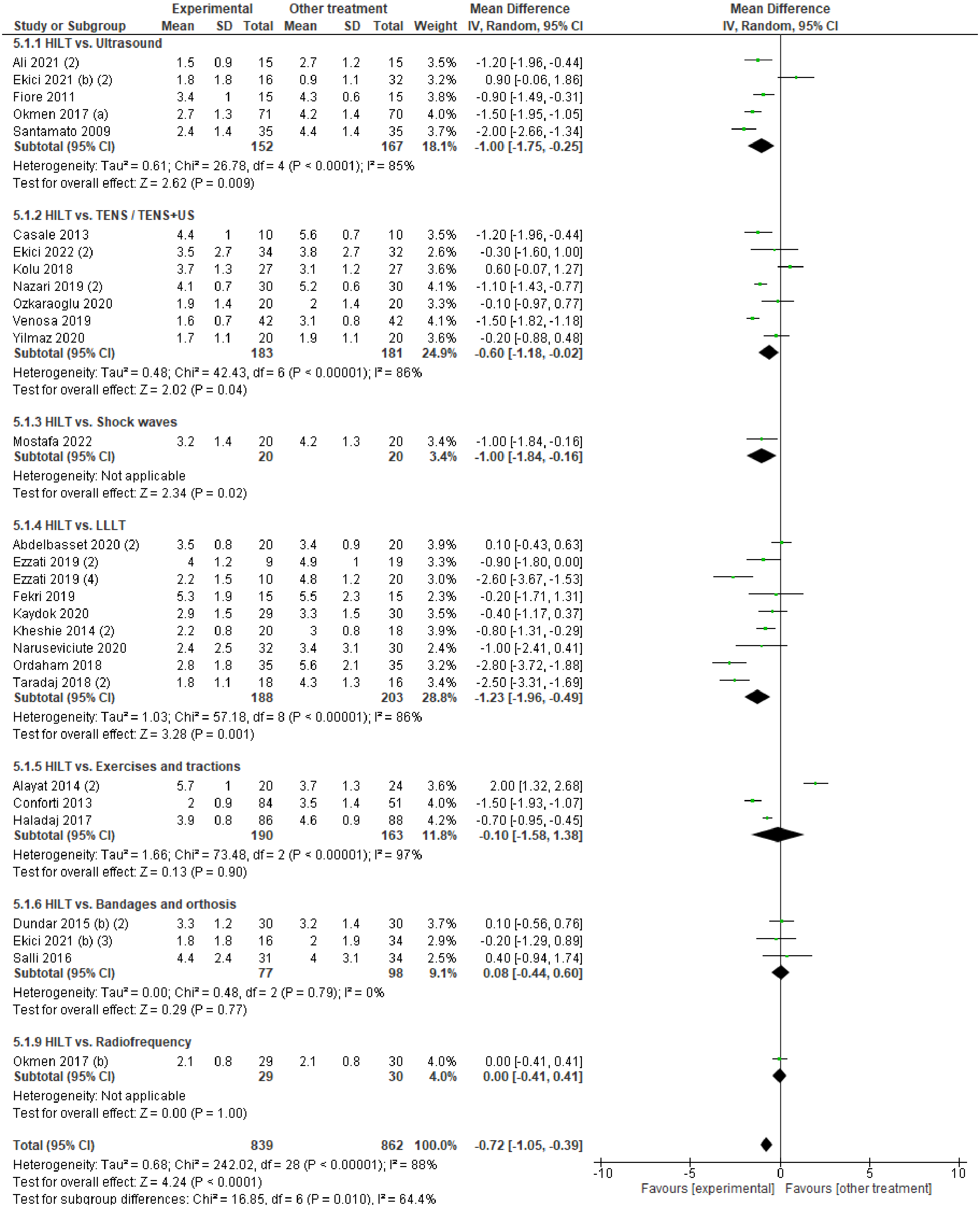

Forest plot for the effect on **functionality** comparing high-intensity laser therapy vs. other treatments. Subgroups analysis by treatment.

| | Experimental | | | Other treatments | | | | Std. Mean Difference |
|---|---|---|---|---|---|---|---|---|
| **Study or Subgroup** | **Mean** | **SD** | **Total** | **Mean** | **SD** | **Total** | **Weight** | **IV, Random, 95% CI** |
| **5.2.1 HILT vs. Ultrasound** | | | | | | | | |
| Ali 2021 (2) | 6.9 | 6.4 | 15 | 15.6 | 8.6 | 15 | 4.1% | -1.12 [-1.89, -0.34] |
| Ekici 2021 (b) (2) | 39.6 | 32 | 16 | 31.6 | 25 | 32 | 4.3% | 0.29 [-0.32, 0.89] |
| Fiore 2011 | 12.4 | 3.2 | 15 | 15.8 | 2.6 | 15 | 4.1% | -1.13 [-1.91, -0.36] |
| Okmen 2017 (a) | 36.6 | 16.5 | 71 | 55.9 | 18.7 | 70 | 4.6% | -1.09 [-1.44, -0.73] |
| Santamato 2009 | -75.9 | 7 | 35 | -72.1 | 7 | 35 | 4.5% | -0.54 [-1.01, -0.06] |
| **Subtotal (95% CI)** | | | **152** | | | **167** | **21.6%** | **-0.71 [-1.23, -0.18]** |
| Heterogeneity: Tau$^2$ = 0.26; Chi$^2$ = 17.41, df = 4 (P = 0.002); I$^2$ = 77% | | | | | | | | |
| Test for overall effect: Z = 2.64 (P = 0.008) | | | | | | | | |
| **5.2.2 HILT vs. TENS / TENS + US** | | | | | | | | |
| Ekici 2022 (2) | 63.8 | 41.1 | 17 | 45.4 | 31.1 | 32 | 4.3% | 0.52 [-0.08, 1.12] |
| Kolu 2018 | 45.1 | 13 | 27 | 54.5 | 14.6 | 27 | 4.4% | -0.67 [-1.22, -0.12] |
| Nazari 2019 (2) | 24.4 | 1.8 | 30 | 27.4 | 2.2 | 30 | 4.4% | -1.47 [-2.05, -0.90] |
| Ozkaraoglu 2020 | 22.5 | 11.8 | 20 | 13.8 | 10.4 | 20 | 4.3% | 0.77 [0.12, 1.41] |
| Venosa 2019 | 6.7 | 2.1 | 42 | 11.8 | 1.4 | 42 | 4.3% | -2.83 [-3.44, -2.22] |
| Yilmaz 2020 | 23.9 | 10.8 | 20 | 26.7 | 17.1 | 20 | 4.3% | -0.19 [-0.81, 0.43] |
| **Subtotal (95% CI)** | | | **156** | | | **171** | **26.1%** | **-0.65 [-1.70, 0.40]** |
| Heterogeneity: Tau$^2$ = 1.62; Chi$^2$ = 91.89, df = 5 (P < 0.00001); I$^2$ = 95% | | | | | | | | |
| Test for overall effect: Z = 1.21 (P = 0.22) | | | | | | | | |
| **5.2.3 HILT vs. Shock waves** | | | | | | | | |
| Mostafa 2022 | 18.1 | 11.2 | 20 | 32.2 | 14.1 | 20 | 4.3% | -1.09 [-1.75, -0.42] |
| **Subtotal (95% CI)** | | | **20** | | | **20** | **4.3%** | **-1.09 [-1.75, -0.42]** |
| Heterogeneity: Not applicable | | | | | | | | |
| Test for overall effect: Z = 3.18 (P = 0.001) | | | | | | | | |
| **5.2.4 HILT vs. LLLT** | | | | | | | | |
| Abdelbasset 2020 (2) | 18.5 | 7.2 | 20 | 17.8 | 6.4 | 20 | 4.3% | 0.10 [-0.52, 0.72] |
| Kaydok 2020 | 24.2 | 10.8 | 29 | 30.1 | 11.1 | 30 | 4.4% | -0.53 [-1.05, -0.01] |
| Kheshie 2014 (2) | 18.7 | 2.3 | 20 | 23.9 | 2.5 | 18 | 4.1% | -2.12 [-2.94, -1.31] |
| Ordaham 2018 | -59.3 | 20.2 | 35 | -52.8 | 20.5 | 35 | 4.5% | -0.32 [-0.79, 0.16] |
| Taradaj 2018 (2) | 17.9 | 7.1 | 18 | 25.6 | 8 | 16 | 4.2% | -1.00 [-1.72, -0.28] |
| **Subtotal (95% CI)** | | | **122** | | | **119** | **21.5%** | **-0.73 [-1.35, -0.10]** |
| Heterogeneity: Tau$^2$ = 0.40; Chi$^2$ = 21.04, df = 4 (P = 0.0003); I$^2$ = 81% | | | | | | | | |
| Test for overall effect: Z = 2.28 (P = 0.02) | | | | | | | | |
| **5.2.5 HILT vs. Exercises and tractions** | | | | | | | | |
| Alayat 2014 (2) | 19.1 | 3 | 20 | 18.8 | 3.1 | 24 | 4.4% | 0.10 [-0.50, 0.69] |
| Haladaj 2017 | 20.7 | 1.7 | 86 | 33.4 | 4.7 | 88 | 4.5% | -3.56 [-4.04, -3.08] |
| **Subtotal (95% CI)** | | | **106** | | | **112** | **8.8%** | **-1.74 [-5.32, 1.85]** |
| Heterogeneity: Tau$^2$ = 6.62; Chi$^2$ = 88.01, df = 1 (P < 0.00001); I$^2$ = 99% | | | | | | | | |
| Test for overall effect: Z = 0.95 (P = 0.34) | | | | | | | | |
| **5.2.6 HILT vs. Bandage and orthosis** | | | | | | | | |
| Dundar 2015 (b) (2) | 39.8 | 12.2 | 30 | 42.1 | 13.7 | 30 | 4.4% | -0.18 [-0.68, 0.33] |
| Ekici 2021 (b) (3) | 39.6 | 32 | 16 | 22.5 | 17.5 | 34 | 4.3% | 0.73 [0.12, 1.34] |
| Salli 2016 | 37.6 | 15.4 | 31 | 35.5 | 24.2 | 34 | 4.5% | 0.10 [-0.39, 0.59] |
| **Subtotal (95% CI)** | | | **77** | | | **98** | **13.2%** | **0.19 [-0.30, 0.68]** |
| Heterogeneity: Tau$^2$ = 0.11; Chi$^2$ = 5.07, df = 2 (P = 0.08); I$^2$ = 61% | | | | | | | | |
| Test for overall effect: Z = 0.77 (P = 0.44) | | | | | | | | |
| **5.2.8 HILT vs. Radiofrequency** | | | | | | | | |
| Okmen 2017 (b) | 26.2 | 20 | 29 | 31.3 | 19.4 | 30 | 4.4% | -0.26 [-0.77, 0.26] |
| **Subtotal (95% CI)** | | | **29** | | | **30** | **4.4%** | **-0.26 [-0.77, 0.26]** |
| Heterogeneity: Not applicable | | | | | | | | |
| Test for overall effect: Z = 0.98 (P = 0.33) | | | | | | | | |
| **Total (95% CI)** | | | **662** | | | **717** | **100.0%** | **-0.67 [-1.11, -0.23]** |
| Heterogeneity: Tau$^2$ = 1.09; Chi$^2$ = 313.73, df = 22 (P < 0.00001); I$^2$ = 93% | | | | | | | | |
| Test for overall effect: Z = 2.95 (P = 0.003) | | | | | | | | |
| Test for subgroup differences: Chi$^2$ = 12.91, df = 6 (P = 0.04), I$^2$ = 53.5% | | | | | | | | |

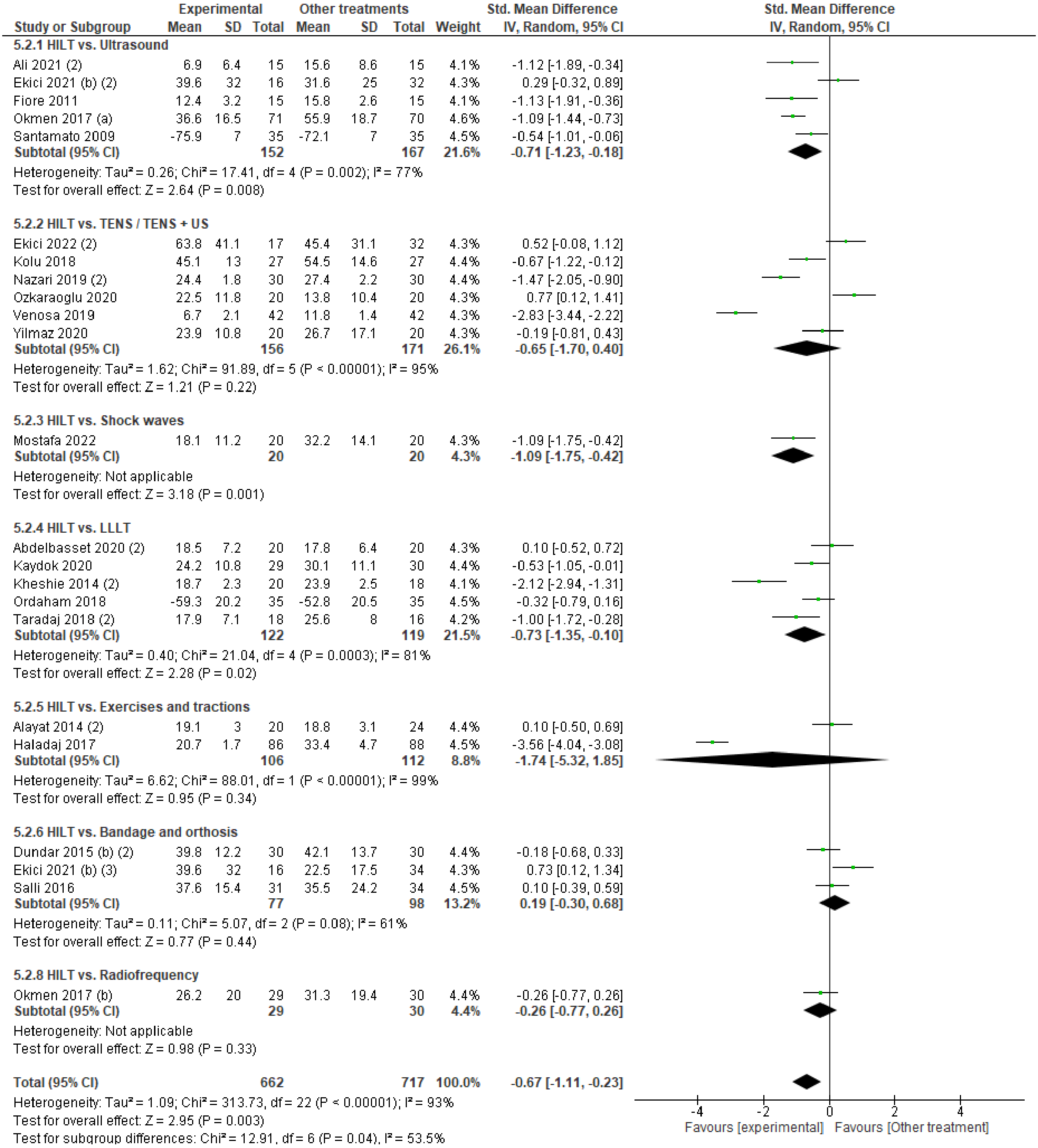

Forest plot for the effect on **VAS pain** comparing high-intensity laser therapy vs. control. Subgroups analysis by follow-up period.

| Study or Subgroup | Experimental Mean | Experimental SD | Experimental Total | Control Mean | Control SD | Control Total | Weight | Mean Difference IV, Random, 95% CI |
|---|---|---|---|---|---|---|---|---|
| **2.1.1 Post inmediate** | | | | | | | | |
| Abdelbasset 2020 (1) | 3.5 | 8 | 20 | 5.9 | 1.8 | 20 | 0.9% | -2.40 [-5.99, 1.19] |
| Alayat 2016 | 1.8 | 0.7 | 30 | 2.8 | 0.8 | 30 | 4.4% | -1.00 [-1.38, -0.62] |
| Alayat 2020 | 1.4 | 0.8 | 25 | 3.6 | 0.7 | 25 | 4.4% | -2.20 [-2.62, -1.78] |
| Ali 2021 (1) | 0.7 | 0.8 | 15 | 2.7 | 1.2 | 15 | 3.9% | -2.00 [-2.73, -1.27] |
| Atan 2020 (1) | 2.9 | 2 | 6 | 6.4 | 2.1 | 10 | 1.9% | -3.50 [-5.56, -1.44] |
| Atan 2020 (2) | 2.9 | 2 | 5 | 7.1 | 1.6 | 10 | 1.9% | -4.20 [-6.21, -2.19] |
| Choi 2017 | 3.4 | 0.8 | 10 | 6.2 | 1.4 | 10 | 3.5% | -2.80 [-3.80, -1.80] |
| Kheshie 2014 (1) | 2.2 | 0.8 | 20 | 3.9 | 0.7 | 15 | 4.3% | -1.70 [-2.20, -1.20] |
| **Subtotal (95% CI)** | | | **131** | | | **135** | **25.2%** | **-2.13 [-2.73, -1.53]** |
| Heterogeneity: Tau² = 0.44; Chi² = 32.71, df = 7 (P < 0.0001); I² = 79% | | | | | | | | |
| Test for overall effect: Z = 7.00 (P < 0.00001) | | | | | | | | |
| **2.1.2 Follow up 3-9 weeks** | | | | | | | | |
| Alayat 2014 (1) | 2.6 | 1.3 | 28 | 3.7 | 1.3 | 24 | 4.0% | -1.10 [-1.81, -0.39] |
| Alayat 2017 | 2.7 | 0.7 | 23 | 4.1 | 0.8 | 22 | 4.4% | -1.40 [-1.84, -0.96] |
| Chen 2017 | 1.2 | 0.9 | 32 | 2 | 1 | 31 | 4.3% | -0.80 [-1.27, -0.33] |
| Dundar 2015 (a) | 2.4 | 1.3 | 38 | 3.9 | 1.3 | 37 | 4.2% | -1.50 [-2.09, -0.91] |
| Dundar 2015 (b) (1) | 3.3 | 1.2 | 30 | 5 | 2.7 | 31 | 3.4% | -1.70 [-2.74, -0.66] |
| Ekici 2021 (a) | 2.6 | 2.4 | 33 | 5.5 | 1.9 | 34 | 3.4% | -2.90 [-3.94, -1.86] |
| Ekici 2021 (b) (1) | 1.8 | 1.8 | 32 | 5.5 | 2 | 34 | 3.6% | -3.70 [-4.62, -2.78] |
| Ekici 2022 (1) | 3.5 | 2.7 | 34 | 5.7 | 1.9 | 34 | 3.3% | -2.20 [-3.31, -1.09] |
| Ezzati 2019 (1) | 4 | 1.2 | 19 | 6.6 | 1.2 | 10 | 3.6% | -2.60 [-3.52, -1.68] |
| Ezzati 2019 (3) | 2.2 | 1.5 | 20 | 6.6 | 1.2 | 10 | 3.5% | -4.40 [-5.39, -3.41] |
| Kim 2015 | 2 | 2.2 | 33 | 2.2 | 2.2 | 33 | 3.4% | -0.20 [-1.26, 0.86] |
| Kim 2016 | 3.1 | 0.7 | 10 | 5.8 | 1.1 | 10 | 3.8% | -2.70 [-3.51, -1.89] |
| Nazari 2019 (1) | 4.1 | 0.7 | 30 | 5.6 | 0.5 | 30 | 4.5% | -1.50 [-1.81, -1.19] |
| Yesil 2020 | 2 | 1.7 | 21 | 2.9 | 2.1 | 21 | 3.2% | -0.90 [-2.06, 0.26] |
| Yilmaz 2022 | 4.9 | 0.8 | 32 | 5.7 | 0.9 | 31 | 4.4% | -0.80 [-1.22, -0.38] |
| **Subtotal (95% CI)** | | | **415** | | | **392** | **56.8%** | **-1.85 [-2.31, -1.38]** |
| Heterogeneity: Tau² = 0.68; Chi² = 104.83, df = 14 (P < 0.00001); I² = 87% | | | | | | | | |
| Test for overall effect: Z = 7.73 (P < 0.00001) | | | | | | | | |
| **2.1.3 Follow up 10-24 weeks** | | | | | | | | |
| Aceituno 2019 | 1.8 | 1.8 | 21 | 2.6 | 2.4 | 22 | 3.0% | -0.80 [-2.06, 0.46] |
| Cantero-Tellez 2019 | 5.6 | 1 | 18 | 5.8 | 1.1 | 17 | 4.0% | -0.20 [-0.90, 0.50] |
| Elsodany 2018 | 1.9 | 0.6 | 30 | 4.9 | 0.7 | 30 | 4.5% | -3.00 [-3.33, -2.67] |
| Nouri 2019 | 3.6 | 2.7 | 20 | 4.2 | 1.6 | 20 | 2.8% | -0.60 [-1.98, 0.78] |
| Taradaj 2018 (1) | 1.8 | 1.1 | 18 | 4.2 | 1.5 | 17 | 3.7% | -2.40 [-3.28, -1.52] |
| **Subtotal (95% CI)** | | | **107** | | | **106** | **18.0%** | **-1.45 [-2.81, -0.08]** |
| Heterogeneity: Tau² = 2.19; Chi² = 63.37, df = 4 (P < 0.00001); I² = 94% | | | | | | | | |
| Test for overall effect: Z = 2.07 (P = 0.04) | | | | | | | | |
| **Total (95% CI)** | | | **653** | | | **633** | **100.0%** | **-1.88 [-2.25, -1.51]** |
| Heterogeneity: Tau² = 0.76; Chi² = 226.77, df = 27 (P < 0.00001); I² = 88% | | | | | | | | |
| Test for overall effect: Z = 10.00 (P < 0.00001) | | | | | | | | |
| Test for subgroup differences: Chi² = 1.04, df = 2 (P = 0.59), I² = 0% | | | | | | | | |

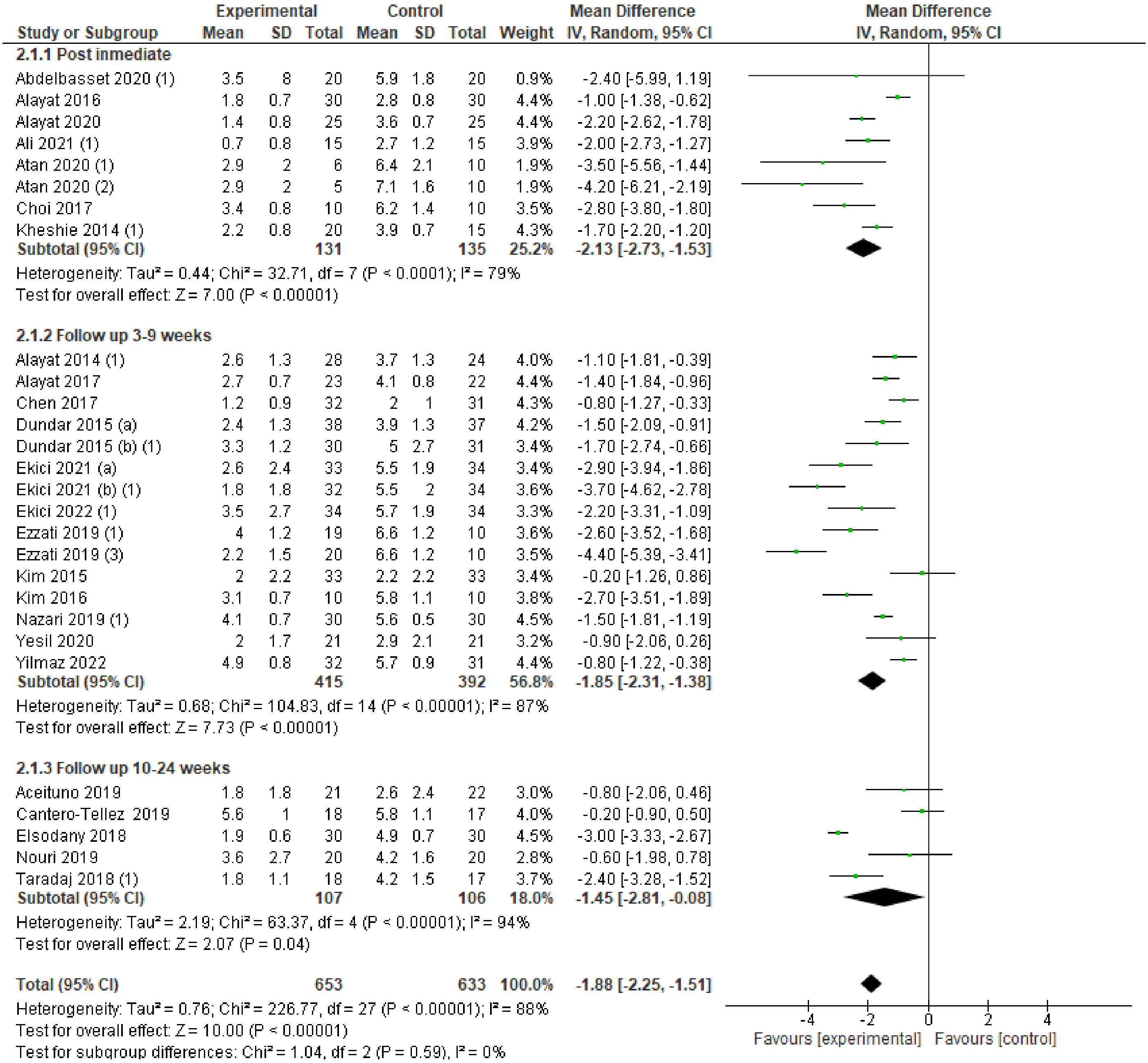

Forest plot for the effect on **VAS pain** comparing high-intensity laser therapy vs. control. Subgroups analysis by dosage.

| Study or Subgroup | Experimental Mean | Experimental SD | Experimental Total | Control Mean | Control SD | Control Total | Weight | Mean Difference IV, Random, 95% CI |
|---|---|---|---|---|---|---|---|---|
| **3.1.5 ≤ 10J/cm$^2$** | | | | | | | | |
| Alayat 2014 (1) | 2.6 | 1.3 | 28 | 3.7 | 1.3 | 24 | 4.3% | -1.10 [-1.81, -0.39] |
| Alayat 2020 | 1.4 | 0.8 | 25 | 3.6 | 0.7 | 25 | 4.8% | -2.20 [-2.62, -1.78] |
| Choi 2017 | 3.4 | 0.8 | 10 | 6.2 | 1.4 | 10 | 3.7% | -2.80 [-3.80, -1.80] |
| Dundar 2015 (a) | 2.4 | 1.3 | 38 | 3.9 | 1.3 | 37 | 4.5% | -1.50 [-2.09, -0.91] |
| Dundar 2015 (b) (1) | 3.3 | 1.2 | 30 | 5 | 2.7 | 31 | 3.6% | -1.70 [-2.74, -0.66] |
| Ekici 2021 (a) | 2.6 | 2.4 | 33 | 5.5 | 1.9 | 34 | 3.6% | -2.90 [-3.94, -1.86] |
| Ekici 2021 (b) (1) | 1.8 | 1.8 | 32 | 5.5 | 2 | 34 | 3.9% | -3.70 [-4.62, -2.78] |
| Ekici 2022 (1) | 3.5 | 2.7 | 34 | 5.7 | 1.9 | 34 | 3.5% | -2.20 [-3.31, -1.09] |
| Ezzati 2019 (3) | 2.2 | 1.5 | 20 | 6.6 | 1.2 | 10 | 3.7% | -4.40 [-5.39, -3.41] |
| Kheshie 2014 (1) | 2.2 | 0.8 | 20 | 3.9 | 0.7 | 15 | 4.7% | -1.70 [-2.20, -1.20] |
| Kim 2016 | 3.1 | 0.7 | 10 | 5.8 | 1.1 | 10 | 4.1% | -2.70 [-3.51, -1.89] |
| Yesil 2020 | 2 | 1.7 | 21 | 2.9 | 2.1 | 21 | 3.4% | -0.90 [-2.06, 0.26] |
| Yilmaz 2022 | 4.9 | 0.8 | 32 | 5.7 | 0.9 | 31 | 4.8% | -0.80 [-1.22, -0.38] |
| **Subtotal (95% CI)** | | | **333** | | | **316** | **52.7%** | **-2.16 [-2.70, -1.62]** |
| Heterogeneity: Tau² = 0.80; Chi² = 88.29, df = 12 (P < 0.00001); I² = 86% | | | | | | | | |
| Test for overall effect: Z = 7.87 (P < 0.00001) | | | | | | | | |
| **3.1.6 > 10 to 50 J/cm$^2$** | | | | | | | | |
| Alayat 2016 | 1.8 | 0.7 | 30 | 2.8 | 0.8 | 30 | 4.8% | -1.00 [-1.38, -0.62] |
| Alayat 2017 | 2.7 | 0.7 | 23 | 4.1 | 0.8 | 22 | 4.8% | -1.40 [-1.84, -0.96] |
| Elsodany 2018 | 1.9 | 0.6 | 30 | 4.9 | 0.7 | 30 | 4.9% | -3.00 [-3.33, -2.67] |
| Ezzati 2019 (1) | 4 | 1.2 | 19 | 6.6 | 1.2 | 10 | 3.9% | -2.60 [-3.52, -1.68] |
| **Subtotal (95% CI)** | | | **102** | | | **92** | **18.4%** | **-1.98 [-3.07, -0.90]** |
| Heterogeneity: Tau² = 1.15; Chi² = 70.62, df = 3 (P < 0.00001); I² = 96% | | | | | | | | |
| Test for overall effect: Z = 3.58 (P = 0.0003) | | | | | | | | |
| **3.1.7 > 50 to 100 J/cm$^2$** | | | | | | | | |
| Nazari 2019 (1) | 4.1 | 0.7 | 30 | 5.6 | 0.5 | 30 | 4.9% | -1.50 [-1.81, -1.19] |
| Taradaj 2018 (1) | 1.8 | 1.1 | 18 | 4.2 | 1.5 | 17 | 4.0% | -2.40 [-3.28, -1.52] |
| **Subtotal (95% CI)** | | | **48** | | | **47** | **8.9%** | **-1.85 [-2.71, -0.99]** |
| Heterogeneity: Tau² = 0.29; Chi² = 3.61, df = 1 (P = 0.06); I² = 72% | | | | | | | | |
| Test for overall effect: Z = 4.22 (P < 0.0001) | | | | | | | | |
| **3.1.8 > 100 to 300 J/cm$^2$** | | | | | | | | |
| Abdelbasset 2020 (1) | 3.5 | 8 | 20 | 5.9 | 1.8 | 20 | 0.9% | -2.40 [-5.99, 1.19] |
| Aceituno 2019 | 1.8 | 1.8 | 21 | 2.6 | 2.4 | 22 | 3.2% | -0.80 [-2.06, 0.46] |
| Ali 2021 (1) | 0.7 | 0.8 | 15 | 2.7 | 1.2 | 15 | 4.3% | -2.00 [-2.73, -1.27] |
| Atan 2020 (1) | 2.9 | 2 | 6 | 6.4 | 2.1 | 10 | 2.0% | -3.50 [-5.56, -1.44] |
| Atan 2020 (2) | 2.9 | 2 | 5 | 7.1 | 1.6 | 10 | 2.0% | -4.20 [-6.21, -2.19] |
| Chen 2017 | 1.2 | 0.9 | 32 | 2 | 1 | 31 | 4.7% | -0.80 [-1.27, -0.33] |
| Nouri 2019 | 3.6 | 2.7 | 20 | 4.2 | 1.6 | 20 | 3.0% | -0.60 [-1.98, 0.78] |
| **Subtotal (95% CI)** | | | **119** | | | **128** | **20.0%** | **-1.73 [-2.61, -0.86]** |
| Heterogeneity: Tau² = 0.83; Chi² = 22.12, df = 6 (P = 0.001); I² = 73% | | | | | | | | |
| Test for overall effect: Z = 3.88 (P = 0.0001) | | | | | | | | |
| **Total (95% CI)** | | | **602** | | | **583** | **100.0%** | **-2.01 [-2.38, -1.64]** |
| Heterogeneity: Tau² = 0.70; Chi² = 199.25, df = 25 (P < 0.00001); I² = 87% | | | | | | | | |
| Test for overall effect: Z = 10.66 (P < 0.00001) | | | | | | | | |
| Test for subgroup differences: Chi² = 0.82, df = 3 (P = 0.85), I² = 0% | | | | | | | | |

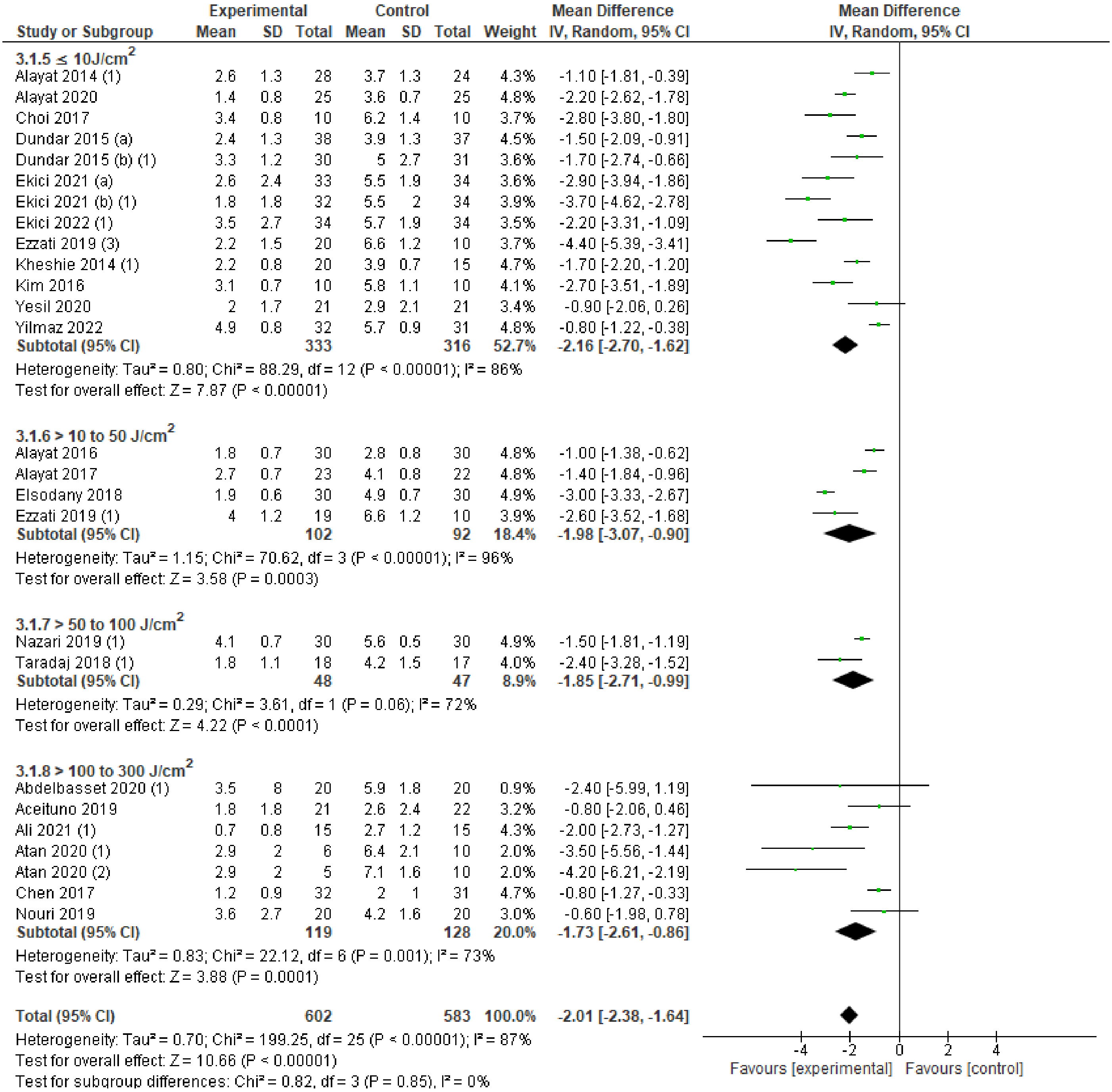

Forest plot for the effect on **VAS pain** comparing high-intensity laser therapy vs. control. Subgroups analysis by location.

| | Experimental | | | Control | | | | Mean Difference |
|---|---|---|---|---|---|---|---|---|
| Study or Subgroup | Mean | SD | Total | Mean | SD | Total | Weight | IV, Random, 95% CI |
| **4.1.1 Shoulder pain** | | | | | | | | |
| Aceituno 2019 | 1.8 | 1.8 | 21 | 2.6 | 2.4 | 22 | 2.9% | -0.80 [-2.06, 0.46] |
| Atan 2020 (1) | 2.9 | 2 | 6 | 6.4 | 2.1 | 10 | 1.8% | -3.50 [-5.56, -1.44] |
| Atan 2020 (2) | 2.9 | 2 | 5 | 7.1 | 1.6 | 10 | 1.9% | -4.20 [-6.21, -2.19] |
| Elsodany 2018 | 1.9 | 0.6 | 30 | 4.9 | 0.7 | 30 | 4.4% | -3.00 [-3.33, -2.67] |
| Kim 2015 | 2 | 2.2 | 33 | 2.2 | 2.2 | 33 | 3.3% | -0.20 [-1.26, 0.86] |
| Yilmaz 2022 | 4.9 | 0.8 | 32 | 5.7 | 0.9 | 31 | 4.3% | -0.80 [-1.22, -0.38] |
| **Subtotal (95% CI)** | | | **127** | | | **136** | **18.5%** | **-1.94 [-3.23, -0.65]** |
| Heterogeneity: Tau² = 2.18; Chi² = 87.42, df = 5 (P < 0.00001); I² = 94% | | | | | | | | |
| Test for overall effect: Z = 2.94 (P = 0.003) | | | | | | | | |
| **4.1.2 Elbow, wrist or thumb pain** | | | | | | | | |
| Ali 2021 (1) | 0.7 | 0.8 | 15 | 2.7 | 1.2 | 15 | 3.8% | -2.00 [-2.73, -1.27] |
| Cantero-Tellez 2019 | 5.6 | 1 | 18 | 5.8 | 1.1 | 17 | 3.9% | -0.20 [-0.90, 0.50] |
| Dundar 2015 (b) (1) | 3.3 | 1.2 | 30 | 5 | 2.7 | 31 | 3.3% | -1.70 [-2.74, -0.66] |
| Ezzati 2019 (1) | 4 | 1.2 | 19 | 6.6 | 1.2 | 10 | 3.5% | -2.60 [-3.52, -1.68] |
| Ezzati 2019 (3) | 2.2 | 1.5 | 20 | 6.6 | 1.2 | 10 | 3.4% | -4.40 [-5.39, -3.41] |
| **Subtotal (95% CI)** | | | **102** | | | **83** | **17.9%** | **-2.16 [-3.51, -0.81]** |
| Heterogeneity: Tau² = 2.16; Chi² = 49.61, df = 4 (P < 0.00001); I² = 92% | | | | | | | | |
| Test for overall effect: Z = 3.14 (P = 0.002) | | | | | | | | |
| **4.1.3 Neck pain** | | | | | | | | |
| Alayat 2016 | 1.8 | 0.7 | 30 | 2.8 | 0.8 | 30 | 4.3% | -1.00 [-1.38, -0.62] |
| Alayat 2020 | 1.4 | 0.8 | 25 | 3.6 | 0.7 | 25 | 4.3% | -2.20 [-2.62, -1.78] |
| Dundar 2015 (a) | 2.4 | 1.3 | 38 | 3.9 | 1.3 | 37 | 4.0% | -1.50 [-2.09, -0.91] |
| **Subtotal (95% CI)** | | | **93** | | | **92** | **12.6%** | **-1.57 [-2.34, -0.79]** |
| Heterogeneity: Tau² = 0.41; Chi² = 17.40, df = 2 (P = 0.0002); I² = 89% | | | | | | | | |
| Test for overall effect: Z = 3.98 (P < 0.0001) | | | | | | | | |
| **4.1.4 Low back pain** | | | | | | | | |
| Abdelbasset 2020 (1) | 3.5 | 0.8 | 20 | 5.9 | 1.8 | 20 | 3.6% | -2.40 [-3.26, -1.54] |
| Alayat 2014 (1) | 2.6 | 1.3 | 28 | 3.7 | 1.3 | 24 | 3.9% | -1.10 [-1.81, -0.39] |
| Chen 2017 | 1.2 | 0.9 | 32 | 2 | 1 | 31 | 4.2% | -0.80 [-1.27, -0.33] |
| Choi 2017 | 3.4 | 0.8 | 10 | 6.2 | 1.4 | 10 | 3.4% | -2.80 [-3.80, -1.80] |
| Taradaj 2018 (1) | 1.8 | 1.1 | 18 | 4.2 | 1.5 | 17 | 3.6% | -2.40 [-3.28, -1.52] |
| **Subtotal (95% CI)** | | | **108** | | | **102** | **18.6%** | **-1.84 [-2.67, -1.01]** |
| Heterogeneity: Tau² = 0.73; Chi² = 24.44, df = 4 (P < 0.0001); I² = 84% | | | | | | | | |
| Test for overall effect: Z = 4.36 (P < 0.0001) | | | | | | | | |
| **4.1.5 Knee pain** | | | | | | | | |
| Alayat 2017 | 2.7 | 0.7 | 23 | 4.1 | 0.8 | 22 | 4.2% | -1.40 [-1.84, -0.96] |
| Kheshie 2014 (1) | 2.2 | 0.8 | 20 | 3.9 | 0.7 | 15 | 4.2% | -1.70 [-2.20, -1.20] |
| Kim 2016 | 3.1 | 0.7 | 10 | 5.8 | 1.1 | 10 | 3.7% | -2.70 [-3.51, -1.89] |
| Nazari 2019 (1) | 4.1 | 0.7 | 30 | 5.6 | 0.5 | 30 | 4.4% | -1.50 [-1.81, -1.19] |
| Nouri 2019 | 3.6 | 2.7 | 20 | 4.2 | 1.6 | 20 | 2.7% | -0.60 [-1.98, 0.78] |
| **Subtotal (95% CI)** | | | **103** | | | **97** | **19.2%** | **-1.64 [-2.04, -1.23]** |
| Heterogeneity: Tau² = 0.11; Chi² = 10.45, df = 4 (P = 0.03); I² = 62% | | | | | | | | |
| Test for overall effect: Z = 8.00 (P < 0.00001) | | | | | | | | |
| **4.1.6 Foot Pain** | | | | | | | | |
| Yesil 2020 | 2 | 1.7 | 21 | 2.9 | 2.1 | 21 | 3.1% | -0.90 [-2.06, 0.26] |
| **Subtotal (95% CI)** | | | **21** | | | **21** | **3.1%** | **-0.90 [-2.06, 0.26]** |
| Heterogeneity: Not applicable | | | | | | | | |
| Test for overall effect: Z = 1.53 (P = 0.13) | | | | | | | | |
| **4.1.7 Temporomandibular pain** | | | | | | | | |
| Ekici 2021 (a) | 2.6 | 2.4 | 33 | 5.5 | 1.9 | 34 | 3.3% | -2.90 [-3.94, -1.86] |
| Ekici 2021 (b) (1) | 1.8 | 1.8 | 32 | 5.5 | 2 | 34 | 3.5% | -3.70 [-4.62, -2.78] |
| Ekici 2022 (1) | 3.5 | 2.7 | 34 | 5.7 | 1.9 | 34 | 3.2% | -2.20 [-3.31, -1.09] |
| **Subtotal (95% CI)** | | | **99** | | | **102** | **10.0%** | **-2.98 [-3.84, -2.12]** |
| Heterogeneity: Tau² = 0.31; Chi² = 4.26, df = 2 (P = 0.12); I² = 53% | | | | | | | | |
| Test for overall effect: Z = 6.80 (P < 0.00001) | | | | | | | | |
| **Total (95% CI)** | | | **653** | | | **633** | **100.0%** | **-1.90 [-2.26, -1.53]** |
| Heterogeneity: Tau² = 0.75; Chi² = 228.92, df = 27 (P < 0.00001); I² = 88% | | | | | | | | |
| Test for overall effect: Z = 10.25 (P < 0.00001) | | | | | | | | |
| Test for subgroup differences: Chi² = 10.89, df = 6 (P = 0.09), I² = 44.9% | | | | | | | | |

Mean Difference
IV, Random, 95% CI

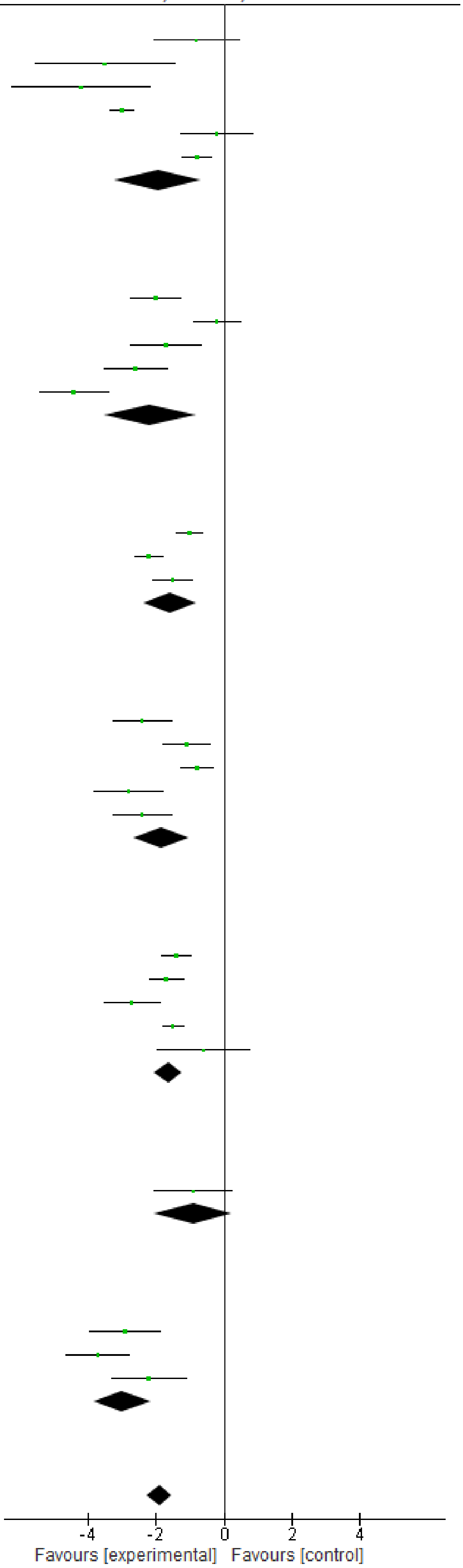

Forest plot for the effect on **functionality** comparing high-intensity laser therapy vs. control **.** Subgroups analysis by follow-up period.

| | Experimental | | | Control | | | | Std. Mean Difference |
|---|---|---|---|---|---|---|---|---|
| **Study or Subgroup** | **Mean** | **SD** | **Total** | **Mean** | **SD** | **Total** | **Weight** | **IV, Random, 95% CI** |
| **2.2.1 Post inmediate** | | | | | | | | |
| Abdelbasset 2020 (1) | 18.5 | 7.2 | 20 | 34.6 | 11.8 | 20 | 4.3% | -1.61 [-2.34, -0.89] |
| Alayat 2016 | 7.8 | 1.7 | 30 | 9.9 | 1.5 | 30 | 4.4% | -1.29 [-1.85, -0.73] |
| Ali 2021 (1) | 5.3 | 6.8 | 15 | 15.6 | 8.6 | 15 | 4.2% | -1.29 [-2.09, -0.50] |
| Atan 2020 (1) | 25.1 | 17 | 6 | 55.5 | 19.6 | 10 | 3.8% | -1.54 [-2.72, -0.35] |
| Atan 2020 (2) | 25.1 | 17 | 5 | 58.6 | 16 | 10 | 3.5% | -1.93 [-3.27, -0.59] |
| Choi 2017 | 19 | 10.6 | 10 | 29.6 | 10.7 | 10 | 4.1% | -0.95 [-1.89, -0.02] |
| Kheshie 2014 (1) | 18.7 | 2.3 | 20 | 29.3 | 2.8 | 15 | 3.7% | -4.10 [-5.32, -2.88] |
| Pekyavas 2016 | 13.2 | 9.5 | 19 | 14.6 | 12.6 | 16 | 4.4% | -0.12 [-0.79, 0.54] |
| **Subtotal (95% CI)** | | | **125** | | | **126** | **32.4%** | **-1.52 [-2.19, -0.85]** |
| Heterogeneity: Tau² = 0.71; Chi² = 34.51, df = 7 (P < 0.0001); I² = 80% | | | | | | | | |
| Test for overall effect: Z = 4.43 (P < 0.00001) | | | | | | | | |
| **2.2.2 Follow 3-9 weeks** | | | | | | | | |
| Alayat 2014 (1) | 15.1 | 4.3 | 28 | 18.8 | 3.1 | 24 | 4.4% | -0.96 [-1.54, -0.38] |
| Alayat 2017 | 21.4 | 1.6 | 23 | 28.5 | 2 | 22 | 4.0% | -3.86 [-4.88, -2.84] |
| Chen 2017 | 3.1 | 2 | 32 | 7.9 | 3.9 | 31 | 4.4% | -1.54 [-2.10, -0.97] |
| Dundar 2015 (a) | 20.3 | 6.2 | 38 | 26.1 | 6.7 | 37 | 4.5% | -0.89 [-1.37, -0.41] |
| Dundar 2015 (b) (1) | 39.8 | 12.2 | 30 | 56 | 27.2 | 31 | 4.5% | -0.75 [-1.28, -0.23] |
| Ekici 2021 (a) | 52.4 | 22.4 | 33 | 51.3 | 24.1 | 34 | 4.5% | 0.05 [-0.43, 0.53] |
| Ekici 2021 (b) (1) | 39.6 | 32 | 32 | 53.3 | 42.1 | 34 | 4.5% | -0.36 [-0.85, 0.13] |
| Ekici 2022 (1) | 63.8 | 41.1 | 34 | 52.8 | 31.7 | 34 | 4.5% | 0.30 [-0.18, 0.77] |
| Kim 2016 | 17.2 | 6.2 | 10 | 31.9 | 6.5 | 10 | 3.8% | -2.22 [-3.38, -1.05] |
| Nazari 2019 (1) | 24.4 | 1.8 | 30 | 36.9 | 1.7 | 30 | 3.5% | -7.05 [-8.45, -5.65] |
| Yesil 2020 | -57.9 | 16.6 | 21 | -65.1 | 19.3 | 21 | 4.4% | 0.39 [-0.22, 1.00] |
| Yilmaz 2022 | -75.8 | 9.4 | 32 | -70.4 | 8.9 | 31 | 4.5% | -0.58 [-1.09, -0.08] |
| **Subtotal (95% CI)** | | | **343** | | | **339** | **51.5%** | **-1.30 [-1.98, -0.62]** |
| Heterogeneity: Tau² = 1.30; Chi² = 172.66, df = 11 (P < 0.00001); I² = 94% | | | | | | | | |
| Test for overall effect: Z = 3.75 (P = 0.0002) | | | | | | | | |
| **2.2.3 Follow 10-24 weeks** | | | | | | | | |
| Aceituno 2019 | 9.9 | 10.7 | 21 | 14.8 | 17.1 | 22 | 4.4% | -0.34 [-0.94, 0.27] |
| Elsodany 2018 | 22 | 1.2 | 30 | 41.4 | 2.6 | 30 | 2.9% | -9.46 [-11.28, -7.63] |
| Nouri 2019 | 16.9 | 14.5 | 20 | 18.9 | 9 | 20 | 4.4% | -0.16 [-0.78, 0.46] |
| Taradaj 2018 (1) | 17.9 | 7.1 | 18 | 19.8 | 6.5 | 17 | 4.4% | -0.27 [-0.94, 0.39] |
| **Subtotal (95% CI)** | | | **89** | | | **89** | **16.1%** | **-2.30 [-4.41, -0.18]** |
| Heterogeneity: Tau² = 4.37; Chi² = 94.33, df = 3 (P < 0.00001); I² = 97% | | | | | | | | |
| Test for overall effect: Z = 2.13 (P = 0.03) | | | | | | | | |
| **Total (95% CI)** | | | **557** | | | **554** | **100.0%** | **-1.49 [-2.00, -0.98]** |
| Heterogeneity: Tau² = 1.44; Chi² = 315.73, df = 23 (P < 0.00001); I² = 93% | | | | | | | | |
| Test for overall effect: Z = 5.72 (P < 0.00001) | | | | | | | | |
| Test for subgroup differences: Chi² = 0.85, df = 2 (P = 0.66), I² = 0% | | | | | | | | |

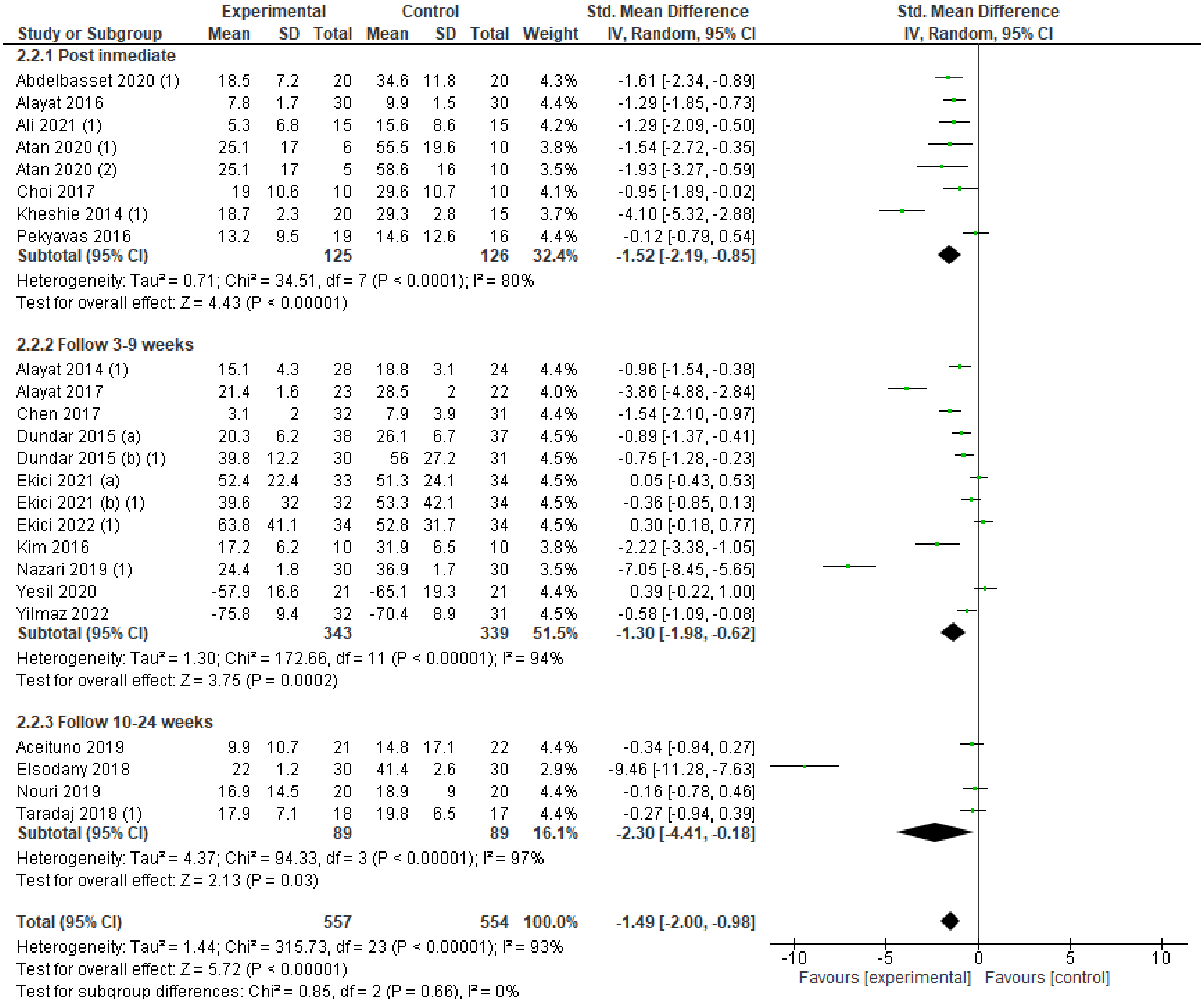

Forest plot for the effect on **functionality** comparing high-intensity laser therapy vs. control **.** Subgroups analysis by dosage.

| Study or Subgroup | Experimental Mean | Experimental SD | Experimental Total | Control Mean | Control SD | Control Total | Weight | Std. Mean Difference IV, Random, 95% CI |
|---|---|---|---|---|---|---|---|---|
| **3.2.1 ≤ 10 J/cm²** | | | | | | | | |
| Alayat 2014 (1) | 15.1 | 4.3 | 28 | 18.8 | 3.1 | 24 | 4.4% | -0.96 [-1.54, -0.38] |
| Choi 2017 | 19 | 10.6 | 10 | 29.6 | 10.7 | 10 | 4.1% | -0.95 [-1.89, -0.02] |
| Dundar 2015 (a) | 20.3 | 6.2 | 38 | 26.1 | 6.7 | 37 | 4.5% | -0.89 [-1.37, -0.41] |
| Dundar 2015 (b) (1) | 39.8 | 12.2 | 30 | 56 | 27.2 | 31 | 4.5% | -0.75 [-1.28, -0.23] |
| Ekici 2021 (a) | 52.4 | 22.4 | 33 | 51.3 | 24.1 | 34 | 4.5% | 0.05 [-0.43, 0.53] |
| Ekici 2021 (b) (1) | 39.6 | 32 | 32 | 53.3 | 42.1 | 34 | 4.5% | -0.36 [-0.85, 0.13] |
| Ekici 2022 (1) | 63.8 | 41.1 | 34 | 52.8 | 31.7 | 34 | 4.5% | 0.30 [-0.18, 0.77] |
| Kheshie 2014 (1) | 18.7 | 2.3 | 20 | 29.3 | 2.8 | 15 | 3.7% | -4.10 [-5.32, -2.88] |
| Kim 2016 | 17.2 | 6.2 | 10 | 31.9 | 6.5 | 10 | 3.8% | -2.22 [-3.38, -1.05] |
| Pekyavas 2016 | 13.2 | 9.5 | 19 | 14.6 | 12.6 | 16 | 4.4% | -0.12 [-0.79, 0.54] |
| Yesil 2020 | -57.9 | 16.6 | 21 | -65.1 | 19.3 | 21 | 4.4% | 0.39 [-0.22, 1.00] |
| Yilmaz 2022 | -75.8 | 9.4 | 32 | -70.4 | 8.9 | 31 | 4.5% | -0.58 [-1.09, -0.08] |
| **Subtotal (95% CI)** | | | **307** | | | **297** | **51.8%** | **-0.71 [-1.16, -0.26]** |
| Heterogeneity: Tau² = 0.52; Chi² = 74.02, df = 11 (P < 0.00001); I² = 85% | | | | | | | | |
| Test for overall effect: Z = 3.08 (P = 0.002) | | | | | | | | |
| **3.2.2 > 10 to 50 J/cm²** | | | | | | | | |
| Alayat 2016 | 7.8 | 1.7 | 30 | 9.9 | 1.5 | 30 | 4.4% | -1.29 [-1.85, -0.73] |
| Alayat 2017 | 21.4 | 1.6 | 23 | 28.5 | 2 | 22 | 4.0% | -3.86 [-4.88, -2.84] |
| Elsodany 2018 | 22 | 1.2 | 30 | 41.4 | 2.6 | 30 | 2.9% | -9.46 [-11.28, -7.63] |
| **Subtotal (95% CI)** | | | **83** | | | **82** | **11.3%** | **-4.77 [-8.51, -1.03]** |
| Heterogeneity: Tau² = 10.54; Chi² = 80.53, df = 2 (P < 0.00001); I² = 98% | | | | | | | | |
| Test for overall effect: Z = 2.50 (P = 0.01) | | | | | | | | |
| **3.2.3 > 50 Jul to 100 J/cm²** | | | | | | | | |
| Nazari 2019 (1) | 24.4 | 1.8 | 30 | 36.9 | 1.7 | 30 | 3.5% | -7.05 [-8.45, -5.65] |
| Taradaj 2018 (1) | 17.9 | 7.1 | 18 | 19.8 | 6.5 | 17 | 4.4% | -0.27 [-0.94, 0.39] |
| **Subtotal (95% CI)** | | | **48** | | | **47** | **7.8%** | **-3.63 [-10.27, 3.01]** |
| Heterogeneity: Tau² = 22.64; Chi² = 73.42, df = 1 (P < 0.00001); I² = 99% | | | | | | | | |
| Test for overall effect: Z = 1.07 (P = 0.28) | | | | | | | | |
| **3.2.4 > 100 to 300 J/cm²** | | | | | | | | |
| Abdelbasset 2020 (1) | 18.5 | 7.2 | 20 | 34.6 | 11.8 | 20 | 4.3% | -1.61 [-2.34, -0.89] |
| Aceituno 2019 | 9.9 | 10.7 | 21 | 14.8 | 17.1 | 22 | 4.4% | -0.34 [-0.94, 0.27] |
| Ali 2021 (1) | 5.3 | 6.8 | 15 | 15.6 | 8.6 | 15 | 4.2% | -1.29 [-2.09, -0.50] |
| Atan 2020 (1) | 25.1 | 17 | 6 | 55.5 | 19.6 | 10 | 3.8% | -1.54 [-2.72, -0.35] |
| Atan 2020 (2) | 25.1 | 17 | 5 | 58.6 | 16 | 10 | 3.5% | -1.93 [-3.27, -0.59] |
| Chen 2017 | 3.1 | 2 | 32 | 7.9 | 3.9 | 31 | 4.4% | -1.54 [-2.10, -0.97] |
| Nouri 2019 | 16.9 | 14.5 | 20 | 18.9 | 9 | 20 | 4.4% | -0.16 [-0.78, 0.46] |
| **Subtotal (95% CI)** | | | **119** | | | **128** | **29.1%** | **-1.12 [-1.66, -0.58]** |
| Heterogeneity: Tau² = 0.36; Chi² = 21.04, df = 6 (P = 0.002); I² = 71% | | | | | | | | |
| Test for overall effect: Z = 4.06 (P < 0.0001) | | | | | | | | |
| **Total (95% CI)** | | | **557** | | | **554** | **100.0%** | **-1.49 [-2.00, -0.98]** |
| Heterogeneity: Tau² = 1.44; Chi² = 315.73, df = 23 (P < 0.00001); I² = 93% | | | | | | | | |
| Test for overall effect: Z = 5.72 (P < 0.00001) | | | | | | | | |
| Test for subgroup differences: Chi² = 6.07, df = 3 (P = 0.11), I² = 50.5% | | | | | | | | |

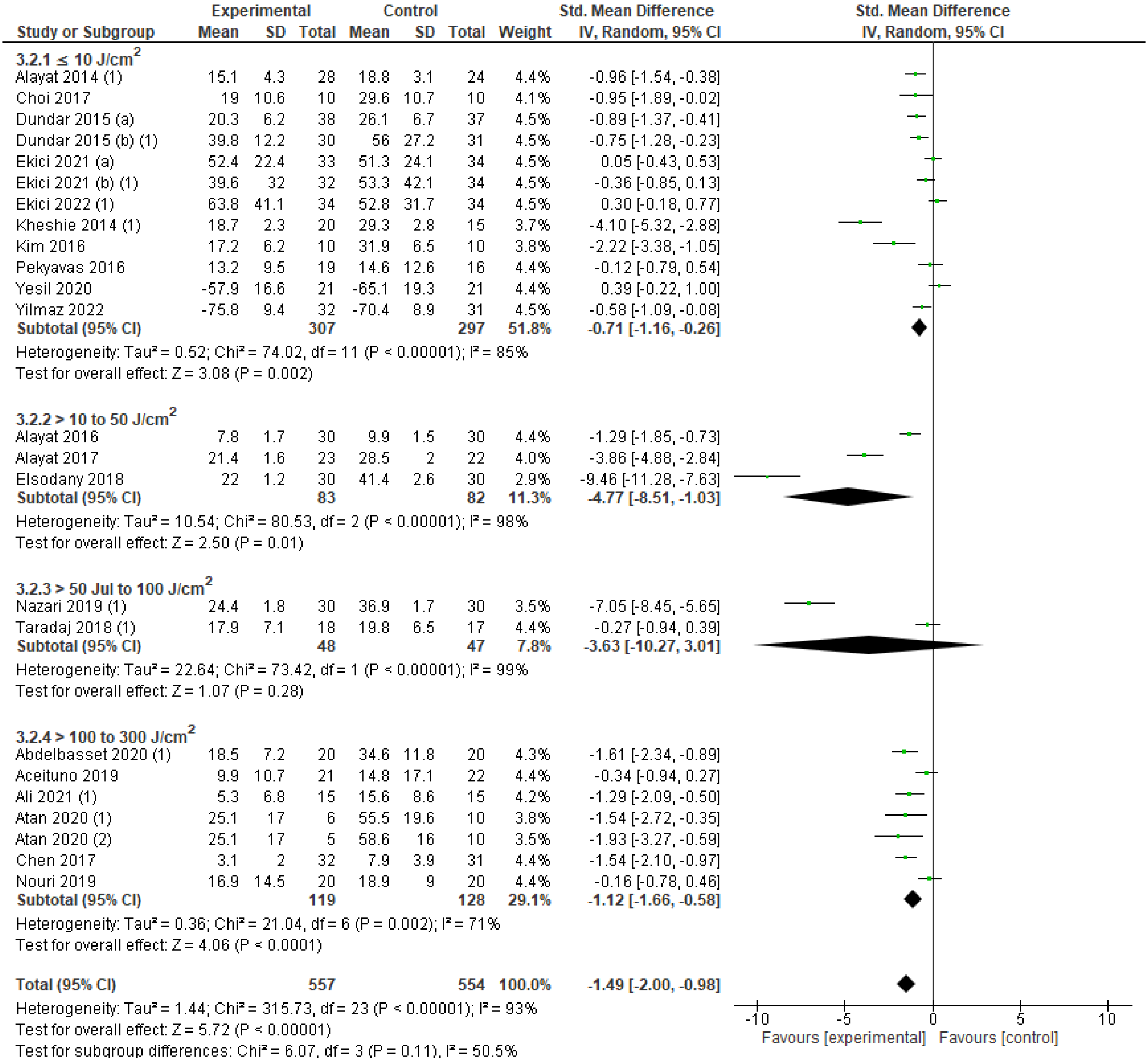

Forest plot for the effect on **functionality** comparing high-intensity laser therapy vs. control. Subgroups analysis by location.

| | Experimental | | | Control | | | | Std. Mean Difference |
|---|---|---|---|---|---|---|---|---|
| Study or Subgroup | Mean | SD | Total | Mean | SD | Total | Weight | IV, Random, 95% CI |
| **4.2.1 Shoulder pain** | | | | | | | | |
| Aceituno 2019 | 9.9 | 10.7 | 21 | 14.8 | 17.1 | 22 | 4.4% | -0.34 [-0.94, 0.27] |
| Atan 2020 (1) | 25.1 | 17 | 6 | 55.5 | 19.6 | 10 | 3.8% | -1.54 [-2.72, -0.35] |
| Atan 2020 (2) | 25.1 | 17 | 5 | 58.6 | 16 | 10 | 3.5% | -1.93 [-3.27, -0.59] |
| Elsodany 2018 | 22 | 1.2 | 30 | 41.4 | 2.6 | 30 | 2.9% | -9.46 [-11.28, -7.63] |
| Pekyavas 2016 | 13.2 | 9.5 | 19 | 14.6 | 12.6 | 16 | 4.4% | -0.12 [-0.79, 0.54] |
| Yilmaz 2022 | -75.8 | 9.4 | 32 | -70.4 | 8.9 | 31 | 4.5% | -0.58 [-1.09, -0.08] |
| **Subtotal (95% CI)** | | | **113** | | | **119** | **23.5%** | **-2.10 [-3.58, -0.62]** |
| Heterogeneity: Tau² = 3.12; Chi² = 97.88, df = 5 (P < 0.00001); I² = 95% | | | | | | | | |
| Test for overall effect: Z = 2.78 (P = 0.005) | | | | | | | | |
| **4.2.2 Elbow, wrist or thumb pain** | | | | | | | | |
| Ali 2021 (1) | 5.3 | 6.8 | 15 | 15.6 | 8.6 | 15 | 4.2% | -1.29 [-2.09, -0.50] |
| Dundar 2015 (b) (1) | 39.8 | 12.2 | 30 | 56 | 27.2 | 31 | 4.5% | -0.75 [-1.28, -0.23] |
| **Subtotal (95% CI)** | | | **45** | | | **46** | **8.7%** | **-0.94 [-1.43, -0.44]** |
| Heterogeneity: Tau² = 0.03; Chi² = 1.23, df = 1 (P = 0.27); I² = 19% | | | | | | | | |
| Test for overall effect: Z = 3.68 (P = 0.0002) | | | | | | | | |
| **4.2.3 Neck Pain** | | | | | | | | |
| Alayat 2016 | 7.8 | 1.7 | 30 | 9.9 | 1.5 | 30 | 4.4% | -1.29 [-1.85, -0.73] |
| Dundar 2015 (a) | 20.3 | 6.2 | 38 | 26.1 | 6.7 | 37 | 4.5% | -0.89 [-1.37, -0.41] |
| **Subtotal (95% CI)** | | | **68** | | | **67** | **9.0%** | **-1.06 [-1.45, -0.67]** |
| Heterogeneity: Tau² = 0.01; Chi² = 1.16, df = 1 (P = 0.28); I² = 14% | | | | | | | | |
| Test for overall effect: Z = 5.33 (P < 0.00001) | | | | | | | | |
| **4.2.4 Low back Pain** | | | | | | | | |
| Abdelbasset 2020 (1) | 18.5 | 7.2 | 20 | 34.6 | 11.8 | 20 | 4.3% | -1.61 [-2.34, -0.89] |
| Alayat 2014 (1) | 15.1 | 4.3 | 28 | 18.8 | 3.1 | 24 | 4.4% | -0.96 [-1.54, -0.38] |
| Chen 2017 | 3.1 | 2 | 32 | 7.9 | 3.9 | 31 | 4.4% | -1.54 [-2.10, -0.97] |
| Choi 2017 | 19 | 10.6 | 10 | 29.6 | 10.7 | 10 | 4.1% | -0.95 [-1.89, -0.02] |
| Taradaj 2018 (1) | 17.9 | 7.1 | 18 | 19.8 | 6.5 | 17 | 4.4% | -0.27 [-0.94, 0.39] |
| **Subtotal (95% CI)** | | | **108** | | | **102** | **21.6%** | **-1.08 [-1.57, -0.59]** |
| Heterogeneity: Tau² = 0.19; Chi² = 10.47, df = 4 (P = 0.03); I² = 62% | | | | | | | | |
| Test for overall effect: Z = 4.31 (P < 0.0001) | | | | | | | | |
| **4.2.5 Knee pain** | | | | | | | | |
| Alayat 2017 | 21.4 | 1.6 | 23 | 28.5 | 2 | 22 | 4.0% | -3.86 [-4.88, -2.84] |
| Kheshie 2014 (1) | 18.7 | 2.3 | 20 | 29.3 | 2.8 | 15 | 3.7% | -4.10 [-5.32, -2.88] |
| Kim 2016 | 17.2 | 6.2 | 10 | 31.9 | 6.5 | 10 | 3.8% | -2.22 [-3.38, -1.05] |
| Nazari 2019 (1) | 24.4 | 1.8 | 30 | 36.9 | 1.7 | 30 | 3.5% | -7.05 [-8.45, -5.65] |
| Nouri 2019 | 16.9 | 14.5 | 20 | 18.9 | 9 | 20 | 4.4% | -0.16 [-0.78, 0.46] |
| **Subtotal (95% CI)** | | | **103** | | | **97** | **19.3%** | **-3.44 [-5.81, -1.07]** |
| Heterogeneity: Tau² = 6.97; Chi² = 107.01, df = 4 (P < 0.00001); I² = 96% | | | | | | | | |
| Test for overall effect: Z = 2.85 (P = 0.004) | | | | | | | | |
| **4.2.6 Foot pain** | | | | | | | | |
| Yesil 2020 | -57.9 | 16.6 | 21 | -65.1 | 19.3 | 21 | 4.4% | 0.39 [-0.22, 1.00] |
| **Subtotal (95% CI)** | | | **21** | | | **21** | **4.4%** | **0.39 [-0.22, 1.00]** |
| Heterogeneity: Not applicable | | | | | | | | |
| Test for overall effect: Z = 1.26 (P = 0.21) | | | | | | | | |
| **4.2.7 Temporomandibular pain** | | | | | | | | |
| Ekici 2021 (a) | 52.4 | 22.4 | 33 | 51.3 | 24.1 | 34 | 4.5% | 0.05 [-0.43, 0.53] |
| Ekici 2021 (b) (1) | 39.6 | 32 | 32 | 53.3 | 42.1 | 34 | 4.5% | -0.36 [-0.85, 0.13] |
| Ekici 2022 (1) | 63.8 | 41.1 | 34 | 52.8 | 31.7 | 34 | 4.5% | 0.30 [-0.18, 0.77] |
| **Subtotal (95% CI)** | | | **99** | | | **102** | **13.5%** | **-0.00 [-0.38, 0.37]** |
| Heterogeneity: Tau² = 0.05; Chi² = 3.62, df = 2 (P = 0.16); I² = 45% | | | | | | | | |
| Test for overall effect: Z = 0.02 (P = 0.99) | | | | | | | | |
| **Total (95% CI)** | | | **557** | | | **554** | **100.0%** | **-1.49 [-2.00, -0.98]** |
| Heterogeneity: Tau² = 1.44; Chi² = 315.73, df = 23 (P < 0.00001); I² = 93% | | | | | | | | |
| Test for overall effect: Z = 5.72 (P < 0.00001) | | | | | | | | |
| Test for subgroup differences: Chi² = 40.08, df = 6 (P < 0.00001), I² = 85.0% | | | | | | | | |

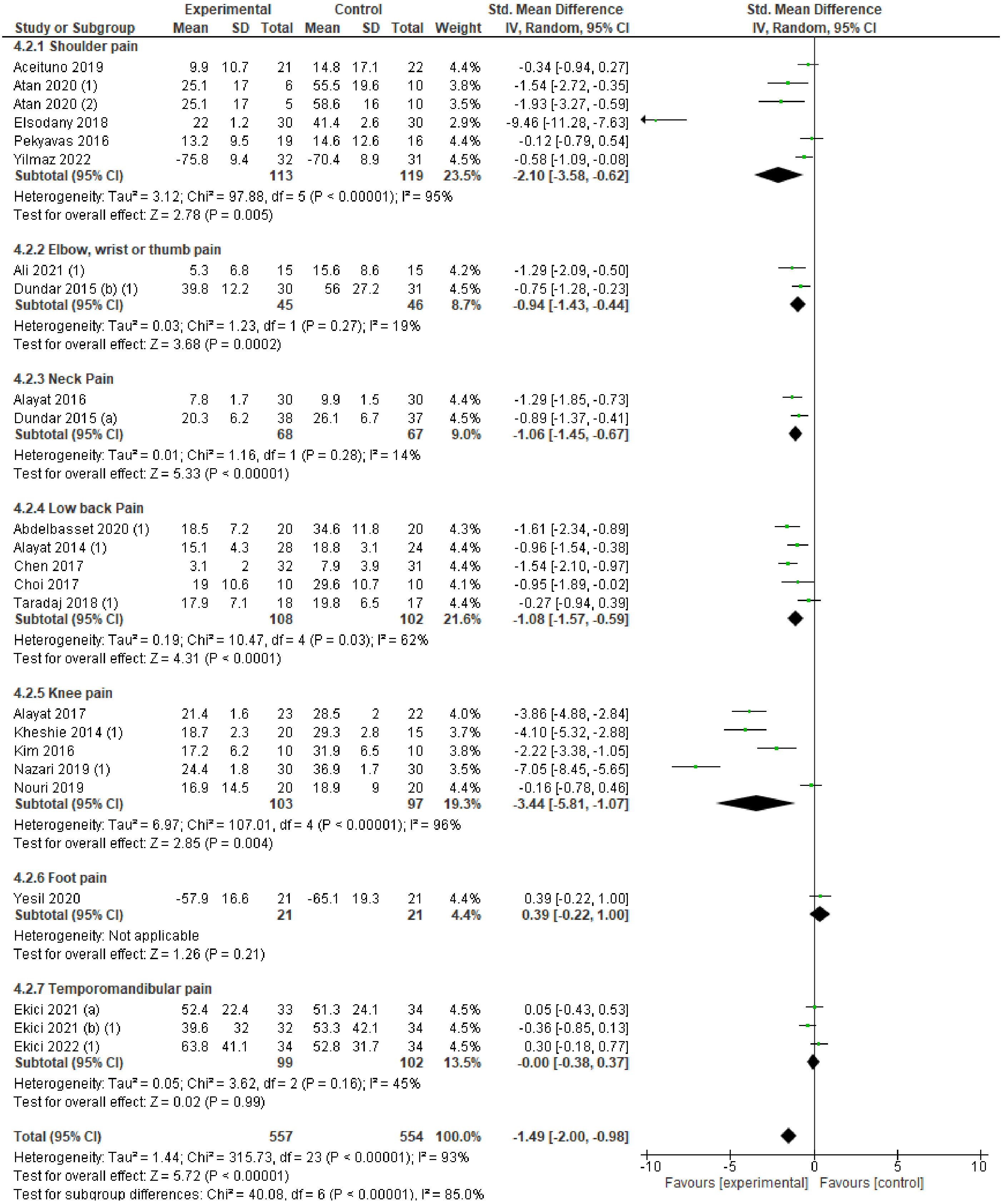

Supplement: Supplementary file 1 [file jcm-12-01479-s001.zip › Suplementary Appendix S3.pdf]
